# Circulating SSEA-1^+^ stem cell-mediated tissue repair in allergic airway inflammation

**DOI:** 10.1007/s00018-022-04366-3

**Published:** 2022-06-07

**Authors:** Chiao-Juno Chiu, Chien-Chia Liao, Yu-Hsiang Hsu, Bor-Luen Chiang

**Affiliations:** 1 Graduate Institute of Clinical Medicine, College of Medicine, National Taiwan University, Taipei, Taiwan; 2 Graduate Institute of Immunology, College of Medicine, National Taiwan University, Taipei, Taiwan; 3 Institute of Clinical Medicine, College of Medicine, National Cheng Kung University, Tainan, Taiwan; 4 Department of Paediatrics, National Taiwan University Hospital, Taipei, Taiwan; 5 Department of Medical Research, National Taiwan University Hospital, No. 7 Chung-Shan South Road, Taipei, 100 Taiwan

**Keywords:** Circulating SSEA-1^+^ cells, Airway inflammation, Tissue repair

## Abstract

Structural changes known as airway remodeling characterize chronic/severe asthma and contribute to lung dysfunction. We previously reported that neonatal SSEA-1^+^ pulmonary stem/progenitor cells (PSCs) ameliorated airway inflammation in asthmatic mice. However, the molecular mechanisms by which endogenous SSEA-1^+^ PSC of adult mice afford beneficial effects in alveolar homeostasis and lung repair after allergen challenge remain incompletely understood. To analyze the expression profile and clarify the biological significance of endogenous adult lung SSEA-1^+^ cells in asthmatic mice. Lung SSEA-1^+^ cells and circulating SSEA-1^+^ cells in peripheral blood were determined by confocal microscopy and cytometric analysis. GFP chimeric mice were used to trace cell lineage in vivo. The roles of circulating SSEA-1^+^ cells were verified in ovalbumin-induced and house dust mite-induced allergic asthmatic models. In asthmatic mice, endogenous lung SSEA-1^+^ cells almost disappeared; however, a unique population of circulating SSEA-1^+^ cells was enriched after the challenge phase. In asthmatic mice, adoptive transfer of circulating SSEA-1^+^ cells had a specific homing preference for the lung in response to inhaled antigen through upregulating CXCR7–CXCL11 chemokine axis. Circulating SSEA-1^+^ cells can transdifferentiate in the alveolar space and ameliorate lung inflammation and structural damage through inhibiting the infiltration of inflammatory cells into peribronchovascular and goblet cell hyperplasia areas, reducing the thickened smooth muscle layers and PAS-positive mucus-containing goblet cells. Reinforcing bone marrow-derived circulating SSEA-1^+^ cells from peripheral blood into lung tissue which create a rescue mechanism in maintaining alveolar homeostasis and tissue repair to mediate lung protection for emergency responses after allergen challenge in asthmatic conditions.

**Supplementary Information:**

The online version contains supplementary material available at 10.1007/s00018-022-04366-3.

## Introduction

Asthma is a heterogeneous airway disease and the major cause of childhood morbidity from chronic diseases [1]. Asthma often begins in early childhood and leads to reversible airway obstruction [11]. The current major treatment for asthma is inhaled corticosteroids to reduce the severity of day-to-day symptoms. However, unwanted local and systemic side effects remain a concern in patients with high-dose and long-term steroid use [27]. Therefore, novel therapeutic treatments for asthma are still needed.

Adult lung tissues have been suggested to host a pool of quiescent stem cells that maintain their number throughout life [36]. Stem cells might differentiate into committed progenitors that terminally differentiate into terminal cell types when activated by extrinsic signals [10, 25]. A previous study showed that BM-derived epithelial progenitor cells migrate and differentiate to repair bleomycin-induced lung damage [12]. Stem cell homing is thought to be critical in tissue regeneration which reflects the capacity of recruitment and homing of stem and progenitor cells to the damaged tissue in need of repair [18].

The efficacy of stem cell homing could be affected by accessory cells such as T lymphocytes which regulate chemokine and cytokine expression. Previous studies showed that CXCL12 and its receptors, CXCR4, and CXCR7 are involved in the homing of hematopoietic stem cells to the bone marrow and mediate the survival as well as the proliferation of human and murine progenitor cells [15, 32]. In addition to CXCL12, CXCR7 can also bind with low affinity to another chemokine CXCL11 (I-TAC) [2]. CXCL11 is a well-established ligand for CXCR3 having pro- and anti-tumorigenic functional capabilities [31]. Therefore, chemokines and their receptors are not only highly promiscuous and pleiotropic, but the same ligand may function in an antagonistic manner depending on its binding to a receptor subtype on the target cells.

We previously [4] revealed that mouse neonatal SSEA-1^+^ pulmonary stem/progenitor cells (PSCs) highly express Clara cell secretory protein (CCSP) and have the ability to self-renew and differentiate into pneumocytes and tracheal epithelial cells. Neonatal SSEA-1^+^ PSCs inhibit LPS-induced thymic stromal lymphopoietin (TSLP) and interleukin (IL)-4-induced eotaxin production in primary lung epithelial cells. Adoptive transfer of neonatal SSEA-1^+^ PSCs has been shown to reduce airway hyperresponsiveness (AHR) and suppress airway damage in OVA-induced asthmatic mice [4]. We have demonstrated that neonatal SSEA-1^+^ PSCs play an immunomodulatory role in the progression of asthma by inhibiting allergen-induced inflammatory responses [4]. However, little is known about the numbers and the distribution of SSEA-1^+^ PSCs in healthy and asthmatic mice. It also remains unclear the role of endogenous SSEA-1^+^ PSCs in adult mice. We aimed to investigate the biological significance of endogenous SSEA-1^+^ PSCs in adult mice in alveolar homeostasis and lung repair after allergen challenge. Therefore, the distributions of SSEA-1^+^ PSCs in lung tissues derived from healthy and asthmatic mice were determined. The frequency and migration route of circulating SSEA-1^+^ cells were also investigated. Finally, the cell fate of circulating SSEA-1^+^ cells was determined using gene expression analysis and fluorescence imaging via confocal microscopy.

## Methods

Materials and methods are provided in the online supplementary information.

## Results

### SSEA-1^+^ PSCs were a rare population in adult mice

**Fig. 1:**
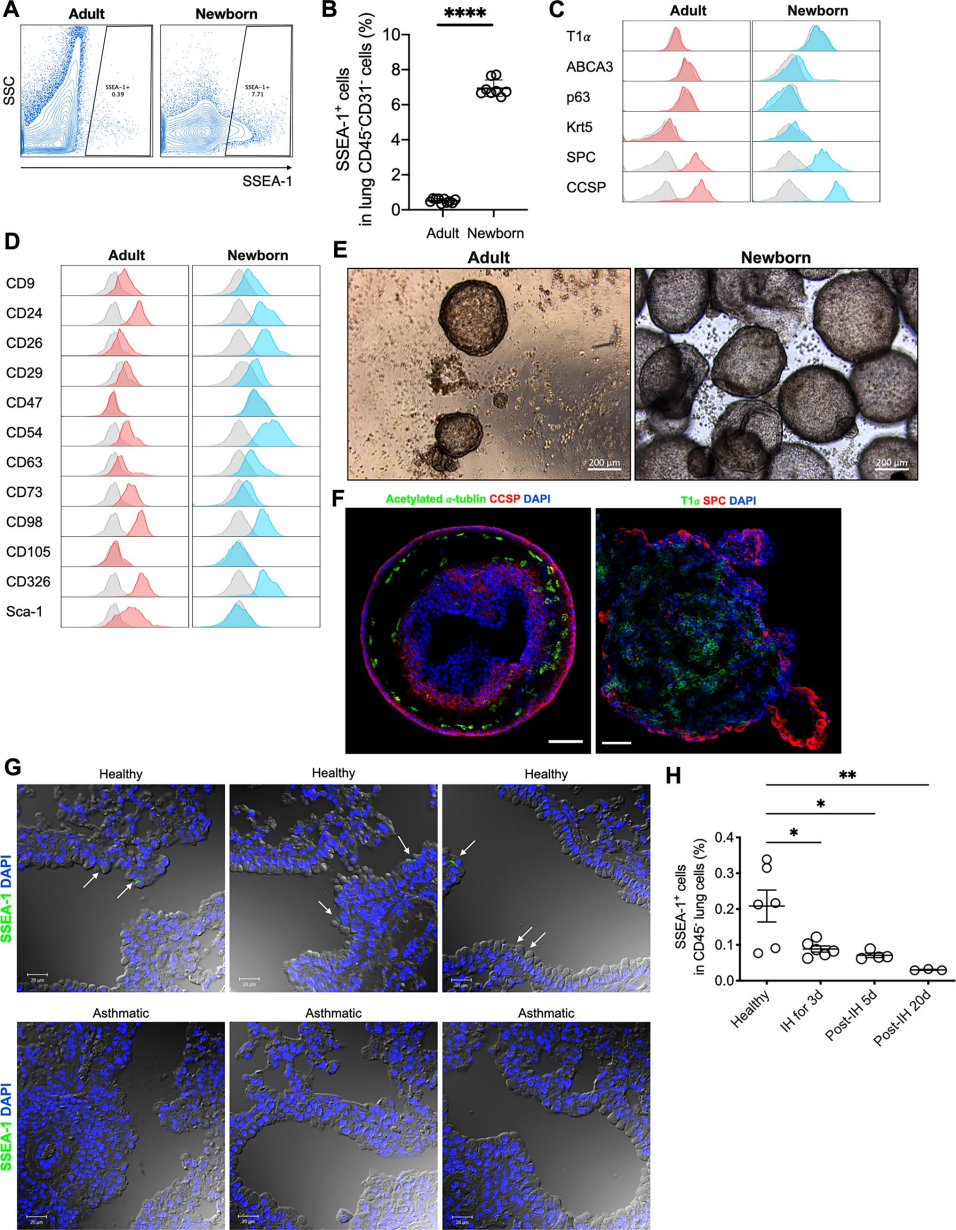
SSEA-1^+^ cells in adult and neonatal mice. **A** Representative FACS plots showing SSEA-1^+^ cells in neonatal- and adult-derived lung cell suspension. Immune and endothelial cells were excluded by gating on CD45^+^ and CD31^+^ cell events. At least 5 × 10^5^ events/sample were acquired. **B** The percentage of CD45^−^CD31^−^ SSEA-1^+^ cell in lung tissue was determined by cytometric analysis. Gray areas represent matched isotype controls. Data are means ± SD and are representative of two independent experiments (adult: *n* = 10, neonatal: *n* = 8). Student's *t* test was performed between adult and new-born groups. ****P < 0.0001 shows statistically significant. **C** Defined cell lineage of lung SSEA-1^+^ cells in adult and neonatal mice by cytometric analysis. Data are representative of two independent experiments. (D) Characteristics of lung SSEA-1^+^ cells in adult and neonatal mice by cytometric analysis. Gray areas represent matched isotype controls. Data are representative of two independent experiments. In (**C**, **D**), FACS analysis of adult and neonatal pulmonary SSEA-1^+^ cells was collected at least 1000 and 10,000 events, respectively. **E** Sphere-forming ability of adult and neonatal SSEA-1^+^ PSC. Data are representative of two independent experiments. **F** Neonatal SSEA-1^+^ PSC-derived spheres contained multipotent stem cells capable of differentiating into different lung cell lineages. Sphere sections were stained with anti-acetylated α-tubulin (green)/CCSP (red) and T1α (green)/SPC (red) to clarify tracheal (left panel)—and pneumocytic (right panel)—cell lineage, respectively; the nuclei were counterstained with DAPI (blue). Bars, 50 μm. Data from one representative experiment of three independent experiments is shown. **G** Adult lung sections were stained with anti-SSEA-1 (green); the nuclei were counterstained with DAPI (blue). The arrows point to the SSEA-1 positive regions. Data are representative of at least three independent experiments. **H** The percentage of pulmonary SSEA-1^+^ cells in lung single cell suspension were determined by cytometric analysis. *IH 3d* inhalation for 3 consecutive days, *post-IH 5d* 5 days after the last inhalation exposure, *post-IH 20d* 20 days after the last inhalation exposure. Data are means ± SEM and are representative of three independent experiments. Statistical significance was determined using ANOVA with Tukey’s multiple-comparison testing between all groups. *P < 0.05 and **P < 0.01 show statistically significant

To investigate the characteristics of SSEA-1^+^ PSCs in adult and neonatal mice, we performed fluorescence-activated cell sorting (FACS) analysis with SSEA-1 antibody and showed that the frequency of SSEA-1^+^ PSCs in adult mice was much lower than that in neonatal mice (Fig. 1A, B). To clarify the difference in lung SSEA-1^+^ cells in adult and neonatal mice, we analyzed lung-associated markers and surface markers on lung SSEA-1^+^ cells. Consistent with the SSEA-1^+^ PSCs in neonatal mice, SSEA-1^+^ PSCs derived from adult mice expressed SPC and CCSP, and these cells were negative for markers of type I pneumocytes (podoplanin; T1α), type II pneumocytes (ATP-binding cassette, class A3; ABCA3), and basal cells (tumor protein p63; p63 and keratin 5; Krt5) (Fig. 1C). Except for that of CD26, CD54, CD73, and Sca-1, surface marker expression of adult SSEA-1^+^ PSCs was compatible with that of those cells in new-born mice (Fig. 1D). To assess the differentiation capacity of SSEA-1^+^ PSCs into specialized somatic cells, enriched pulmonary SSEA-1^+^ single cells from adult mice were embedded in Matrigel and grown in a 3D sphere-formation assay. After 14 days, sphere formation is initiated in neonatal but not in adult mouse-derived pulmonary SSEA-1^+^ cells (Fig. 1E and Supplementary Information Fig. 1). As in our previous findings [4], neonatal mouse-derived pulmonary SSEA-1^+^ PSCs successfully differentiated into pneumocytic and tracheal epithelial cell lineages in these spheres (Fig. 1F). We examined the viability of adult pulmonary SSEA-1^+^ cells using FVS780 staining and observed that the cells were alive in the Matrigel after 14 days of cultivation (Supplementary Information Fig. 2). These data indicated that neonatal and adult mouse-derived pulmonary SSEA-1^+^ cells expressed similar surface- and lineage-associated markers; however, adult mouse-derived pulmonary SSEA-1^+^ cells lost their stem cell-feature sphere-forming ability in vitro.

### Lung-resident SSEA-1^+^ cells were rare in healthy adult mice and asthmatic mice

To clarify the numbers and distribution of lung SSEA-1^+^ cells in healthy and asthmatic mice (Supplementary Information Fig. 3), lung tissue sections and total lung cell suspensions were prepared to detect the expression of SSEA-1. Similar to previous findings [4], immunofluorescence staining of lung tissues revealed that lung SSEA-1^+^ cells resided in the bronchioles and bronchioalveolar–duct junction (BADJ) of neonatal mice, but these cells were rare and resided only in the BADJ in adult mice (Fig. 1G; upper panel; Supplementary Information Fig. 4). Moreover, staining for SSEA-1^+^ cells was almost negative in the lung tissues after induction of airway inflammation in asthmatic mice (Fig. 1G; lower panel). FACS analysis showed that lung SSEA-1^+^ cells in healthy adult mice accounted for approximately 0.2% of the total lung suspension cells; however, this population was significantly depleted a few days post-challenge in asthmatic mice (Fig. 1H), which is consistent with the immunofluorescence imaging results (Fig. 1G).

### Circulating SSEA-1^+^ cells were enriched in murine asthmatic models

**Fig. 2:**
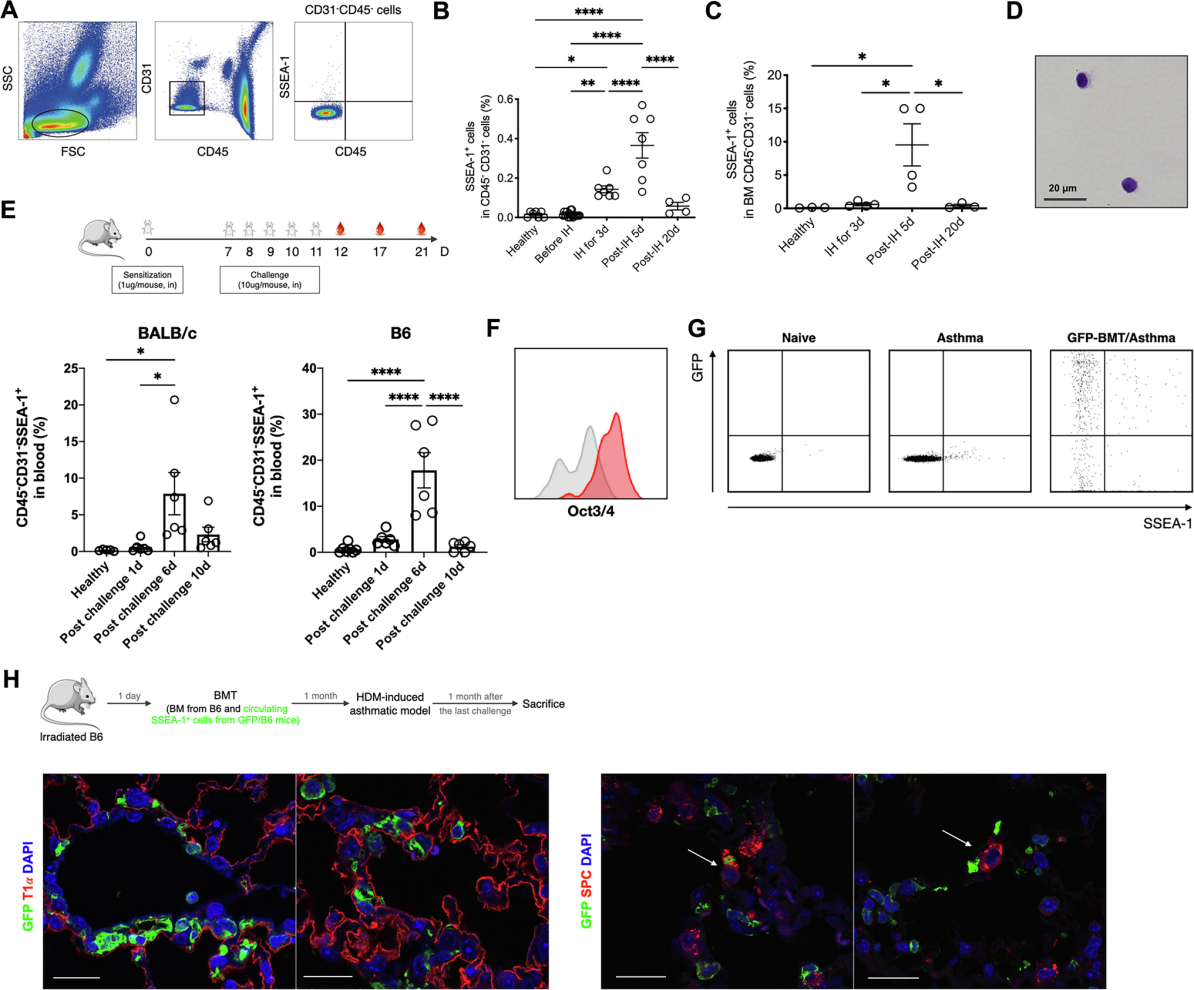
Circulating SSEA-1^+^ cells were increased and transdifferentiated into alveolar space after the challenge stage of murine asthmatic model. **A** Flow cytometric analyses of SSEA-1^+^ cells in peripheral blood. Representative flow cytometric profiles of SSEA-1^+^ cells in peripheral blood are identified. Hematopoietic cells and endothelial cells expressing CD45 and CD31, respectively, were excluded. The percentage of SSEA-1^+^ cells in (**B**) peripheral blood and **C** bone marrow was determined by cytometric analysis. At least 3 × 10^5^ CD45^−^CD31^−^ events/sample were acquired. Data are means ± SEM and are representative of three independent experiments. *IH* inhalation. Statistical significance was determined using ANOVA with Tukey’s multiple-comparison testing between all groups. *P < 0.05, **P < 0.01, and ****P < 0.0001 show statistically significant. **D** H&E staining of purified circulating SSEA-1^+^ cells. **E** The percentage of SSEA-1^+^ cells in peripheral blood derived from HDM-induced asthmatic mice were determined by cytometric analysis. Data are means ± SEM and are representative of three independent experiments. At least 2 × 10^5^ CD45^−^CD31^−^ events/sample were acquired. Statistical significance was determined using ANOVA with Tukey’s multiple-comparison testing between all groups. *P < 0.05 and ****P < 0.0001 show statistically significant. **F** Oct3/4 expression of circulating SSEA-1^+^ cells by FACS analysis. Gray areas represent matched isotype controls. At least 500 events/sample were acquired. **G** Host mice were received BMT from GFP-reporter mice and thereafter induced into HDM-induced asthmatic model. GFP signal in circulating SSEA-1 cells was determined by FACS analysis 1 day after the last HDM exposure. At least 4 × 10^4^ events/sample were acquired. **H** GFP-positive cells in lung tissue-derived from HDM-induced asthmatic mice. Adult lung sections were stained with anti-GFP (green), T1α (red), and SPC (red) antibodies; the nuclei were counterstained with DAPI (blue). Scale bar = 20 μm. Data are representative of at least five independent experiments

To examine whether circulating SSEA-1^+^ cells were present in the bloodstream, we used the surface markers CD45 and CD31 to exclude the haematopoietic cell and endothelial cell populations and then detected the expression of circulating SSEA-1 in the peripheral blood (Fig. 2A). FACS analysis showed that circulating SSEA-1^+^ cells were very difficult to be detected in the peripheral blood of healthy adult mice (Fig. 2B). Interestingly, circulating SSEA-1^+^ cells were detectable in ovalbumin (OVA)-induced asthmatic mice, and the number of circulating SSEA-1^+^ cells was significantly increased in the peripheral blood and bone marrow (BM) at 5 days post-challenge in asthmatic mice (Fig. 2B, C). Microscope-acquired images showed that these circulating SSEA-1^+^ cells were relatively small in size (7.6 ± 0.5 µm) and frequently round in shape (Fig. 2D). In addition, we further observed that the similar phenomenon of the circulating SSEA-1^+^ cells was increased in house dust mite (HDM)-exposed BALB/c and C57BL/6 mice, which was the model used to induce features of clinical asthma. FACS analysis showed that there was a higher frequency of circulating SSEA-1^+^ cells after the last challenge in HDM-induced asthmatic mice than in OVA-induced asthmatic mice (Fig. 2E).

### Circulating SSEA-1^+^ cells were derived from bone marrow and transdifferentiated into pneumocytes of lung tissue

Due to circulating SSEA-1^+^ cells were enriched in the peripheral blood and BM after challenge in asthmatic mice, which attracted us to investigate the physiological properties of these cells. We first explore the stem cell properties of this unique cell population, and we performed an FACS-based screen using a collection of monoclonal antibodies directed against cell surface markers and showed that circulating SSEA-1^+^ cells were negative for mesenchymal stem cell (MSC)- and epithelial cell-associated markers (CD44, CD73, CD105, CD326, and Sca-1) (Supplementary Information Fig. 5A). BM-derived MSCs and primary lung cells were used as positive controls for the antibodies (Supplementary Information Fig. 5B, C). To compare with lung-derived and circulating SSEA-1^+^ cells, we performed FACS to screen the cell surface and lung lineage markers listed in the Fig. 1C, D. The results showed that circulating SSEA-1^+^ cells were positive for CD9, CD24, CD54, CD63, and CD98, dimly express CD26, CD29 and CD73, and negative for CD47, CD105, CD326, Sca-1 and all lung lineage specific markers (T1α, ABCA3, p63, Krt5, SPC, and CCSP) (Supplementary Information Fig. 6). In addition, FACS analysis showed that Oct3/4 expressed in circulating SSEA-1^+^ cells (Fig. 2F). These data suggested that circulating SSEA-1^+^ cells are not derived from mesenchymal, haematopoietic, endothelial, or epithelial cell lineages. Moreover, lung-resident SSEA-1^+^ cells and circulating SSEA-1^+^ cells are different cell populations. Circulating SSEA-1^+^ cells are a unique cell population and express key pluripotent stem cell marker of pluripotency.

To validate the origin of circulating SSEA-1^+^ cells, C57BL/6 mice were transplanted with BM cells from GFP-reporter mice (abbreviated as GFP/BMT mice; Supplementary Information Fig. 7). After 5 weeks of recovery, the GFP/BMT mice were sensitized with HDM. One day after the last HDM challenge, the GFP signal in circulating SSEA-1^+^ cells was determined. FACS analysis showed that almost all circulating SSEA-1^+^ cells in GFP/BMT asthmatic mice were positive for GFP (Fig. 2G), which suggested that circulating SSEA-1^+^ cells found in the blood come from BM.

To further explore the differentiation capacity and cell fate of circulating SSEA-1^+^ cells, we established irradiated chimaeras by transplanting C57BL/6 mice with BM cells from GFP-reporter C57BL/6 mice or BM cells from C57BL/6 mice along with circulating SSEA-1^+^ cells from GFP-reporter mice. Thereafter, chimaeras underwent HDM-induced asthmatic model establishment, and mice were sacrificed 1 month after the last allergen challenge. Confocal fluorescence microscopy for GFP signal was used to characterize circulating SSEA-1^+^ cells in the lung tissue of transplant recipients. We observed that BM-derived circulating SSEA-1^+^ cells migrated to the lung tissue and subsequently differentiated into T1α^+^ type I and SPC^+^ type II pneumocytes in the alveolar space (Fig. 2H).

### Circulating SSEA-1^+^ cells migrated into the lung of asthmatic mice in response to inhaled antigen

**Fig. 3:**
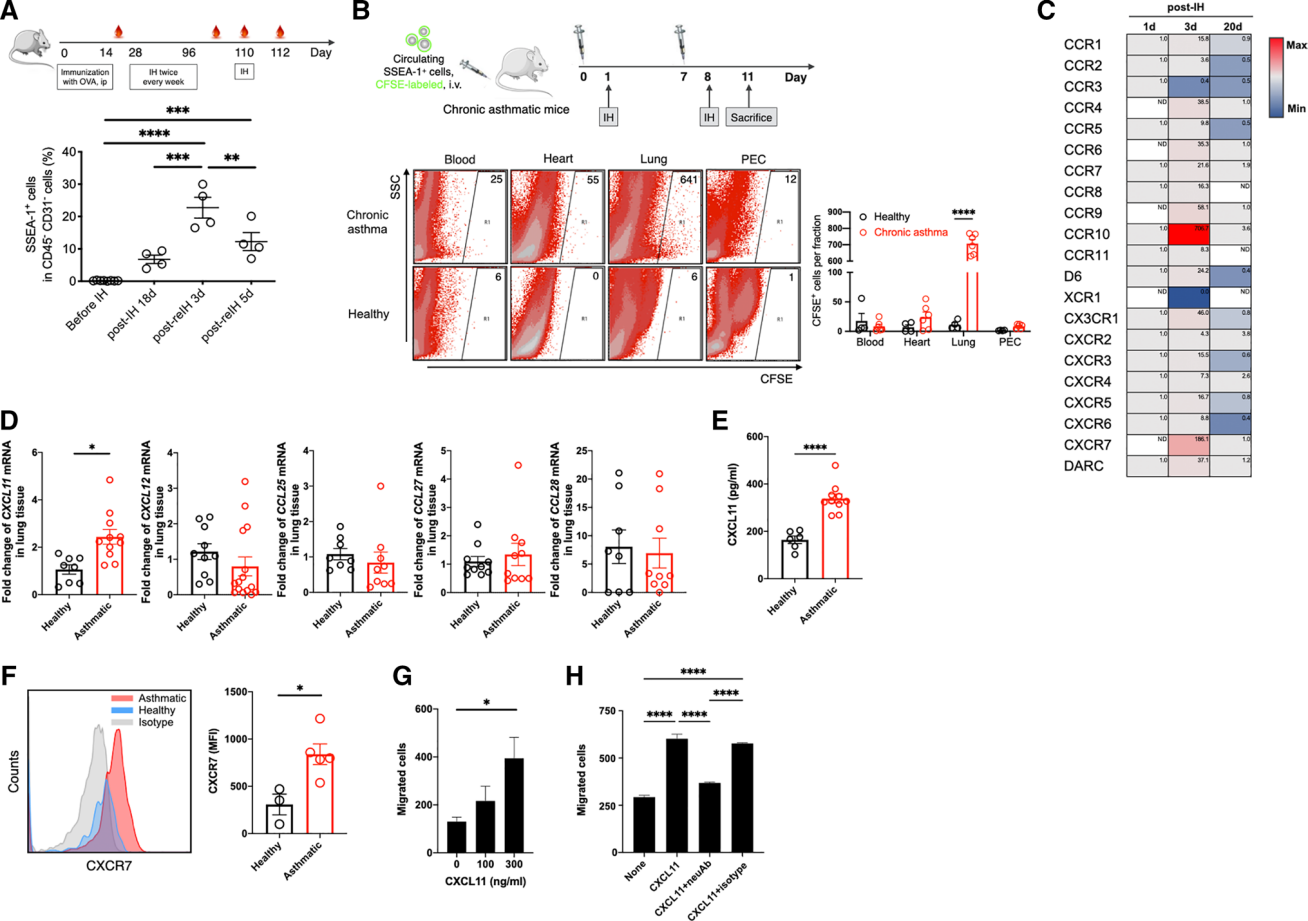
Circulating SSEA-1^+^ cells homed efficiently into the lung in response to inhaled antigen in asthmatic mice. **A** The percentage of circulating SSEA-1^+^ cells in OVA-induced chronic asthmatic mice were determined by cytometric analysis. Data are means ± SEM and are representative of three independent experiments. *IH* inhalation. At least 2 × 10^5^ CD45^−^CD31^−^ events/sample were acquired. Statistical significance was determined using ANOVA with Tukey’s multiple-comparison testing between all groups. **P < 0.01, ***P < 0.001, and ****P < 0.0001 show statistically significant. **B** CFSE-labeled circulating SSEA-1^+^ cells were adoptively transferred into chronic asthmatic recipient mice, followed by two inhaled OVA challenges. Twenty-four hours after the last challenge, the mice were sacrificed and CFSE^+^ cells in blood, heart, lung, and peritoneal exudate cells (PEC) were analyzed by FACS. Gates indicate circulating SSEA-1^+^ cells recovered from various organs, expressed as number of detected CFSE^+^ cells per 5 × 10^5^ cells acquired by FACS. Data are means ± SEM. Student's *t* test was performed between healthy and chronic asthma groups. ****P < 0.0001 shows statistically significant. **C** Heatmap comparing the expression of 21 chemokine receptors in the circulating SSEA-1^+^ cells derived from asthmatic mice. Data are representative of two independent experiments. **D** Chemokine expression levels in lung tissue were determined using RTQ-PCR with specific primers. The mRNA levels were normalized using the housekeeping gene *gapdh*. Data are mean ± SEM. Student's *t* test was performed between healthy and asthma groups. *P < 0.05 shows statistically significant. **E** Levels of CXCL11 in lung tissue analyzed by ELISA. Student's *t* test was performed between healthy and asthma groups. ****P < 0.0001 shows statistically significant. **F** CXCR7 expression of circulating SSEA-1^+^ cells derived from healthy and asthmatic mice. At least 500 events/sample were acquired. Gray areas represent matched isotype controls. Data are means ± SEM and are representative of two independent experiments. Student's *t* test was performed between healthy and asthma groups. *P < 0.05 shows statistically significant. **G** Circulating SSEA-1^+^ cells’ migration in response to CXCL11. Circulating SSEA-1^+^ cells (insert of the transwell) were cultured in the presence of various concentrations of CXCL11 (outer of the transwell) for 4 h. Statistical significance was determined using ANOVA with Tukey’s multiple-comparison testing between all groups. *P < 0.05 shows statistically significant. **H** For CXCL11 neutralization assay, 300 ng/ml CXCL11 were preincubated with 3 μg/ml of anti-CXCL11 neutralization antibody or isotype-matched antibody for 30 min on ice. Then, circulating SSEA-1^+^ cells (insert of the transwell) were cultured in the presence of 300 ng/ml CXCL11 or CXCL11-antibody mixed treatment (lower part of the transwell) for 4 h. The number of migrated cells across membrane in the lower part of the transwell was counted by flow cytometry. Data are means ± SEM and are representative of two independent experiments. Statistical significance was determined using ANOVA with Tukey’s multiple-comparison testing between all groups. ****P < 0.0001 shows statistically significant

To further clarify the biological role of circulating SSEA-1^+^ cells in asthma, we generated a long-term chronic OVA-induced asthmatic murine model. FACS analysis showed that circulating SSEA-1^+^ cells were almost undetectable in mice before OVA inhalation and remarkably enriched in OVA-induced chronic asthmatic mice after long-term OVA inhalation (18 days after the last inhalation) (Fig. 3A). We hypothesized that circulating SSEA-1^+^ cells might play a role in lung repair after allergen challenge. To explore this possibility, chronic asthmatic mice were rechallenged with OVA aerosols via inhalation, and we found that the numbers of circulating SSEA-1^+^ cells were increased more significantly in the peripheral blood at 3 days post-rechallenge in asthmatic mice (Fig. 3A). These data indicated that circulating SSEA-1^+^ cells were enriched after allergen rechallenge in chronic asthmatic conditions. In addition, this circulating SSEA-1^+^ cell population was significantly enriched in the long-term chronic asthmatic model compared with the short-term acute asthmatic model (0.2% and 18%, respectively) (Figs. 2B, 3A).

Our data raised the possibility that reinforcement of SSEA-1^+^ cells from peripheral blood to lung tissue might be a rescue mechanism for emergency responses after allergen challenge. To prove this hypothesis, circulating SSEA-1^+^ fractions isolated from asthmatic mice were collected and labeled with carboxyfluorescein succinimidyl ester (CFSE), a fluorescent cell-staining dye, for further adoptive transfer studies. These cells were intravenously delivered into mice after long-term OVA aerosol exposure (Fig. 3B). FACS analysis showed that CFSE-labeled circulating SSEA-1^+^ cells specifically homed to the lung in response to inhaled antigen in chronic asthmatic mice. However, we did not observe this phenomenon in untreated healthy mice (Fig. 3B).

### Circulating SSEA-1^+^ cells homed to lung tissue through the CXCR7–CXCL11 axis and enhanced tissue repair-associated gene expression

The lung is an important tertiary lymphoid organ, with constant trafficking of cells through the lung in both health and disease [26]. Chemokines and their receptors play a major role in directing the circulating cells into the lung tissue, often in an organ-specific manner [3]. To reveal the mechanism of how circulating SSEA-1^+^ cells migrate to the lung, we next investigated the expression of chemokine receptors on circulating SSEA-1^+^ cells. RT-QPCR showed that the chemokine receptors *CCR10*, *CXCR7*, and *CCR9* were the top three upregulated markers on circulating SSEA-1^+^ cells a few days post-challenge in chronic asthmatic mice (Fig. 3C). CCL25, CCL27/CCL28, and CXCL11/CXCL12 are the functional ligands for CCR9, CCR10, and CXCR7, respectively [31, 35, 37]. RT-QPCR showed that *CXCL11* expression was significantly increased in the lung tissues in asthmatic mice compared to that in healthy mice (Fig. 3D). Consistent with the RT-QPCR data, lung CXCL11 levels were significantly increased in asthmatic mice compared to healthy mice (Fig. 3E). In addition, FACS analysis also showed that CXCR7 protein expression was increased on circulating SSEA-1^+^ cells in asthmatic mice compared with in healthy mice (Fig. 3F). In an ex vivo study, we demonstrated that CXCL11 induced the migration of circulating SSEA-1^+^ cells in a dose-dependent manner (Fig. 3G). To further validate the crucial role of the CXCR7–CXCL11 axis for circulating SSEA-1^+^ cell migration, we used the CXCL11 neutralization antibody to verify the mechanism underlying the migration of circulating SSEA-1^+^ cells. CXCL11 treatment significantly increased the migration of circulating SSEA-1^+^ cells, and the effect was inhibited by the CXCL11 neutralizing antibody treatment (Fig. 3H). Taken together, these data suggested that bone marrow-derived circulating SSEA-1^+^ cells home to the lung through the CXCR7–CXCL11 axis.

To investigate the cell fate of migrated circulating SSEA-1^+^ cells in the lung tissues of asthmatic mice, we collected the whole lung tissue from donor mice and labeled with the same clone of anti-SSEA-1 antibody (Supplementary Information Fig. 8A). FACS analysis showed that circulating SSEA-1^+^ cells migrated to the inflamed lung tissue and loss of SSEA-1 expression 30 days after adoptive transfer (Supplementary Information Fig. 8B). Otherwise, low frequency and retained SSEA-1 expression of transferred circulating SSEA-1^+^ cells can be detected in heart, peritoneal cavity, kidney, and liver. Because circulating SSEA-1^+^ cells migrated to lung and loss of SSEA-1 expression, which raises the possibility that asthmatic mice-derived circulating SSEA-1^+^ cells might initiate differentiation and response to lung repair.

### HGF expressing asthmatic circulating SSEA-1^+^ cells regulated lung development-associated genes

**Fig. 4:**
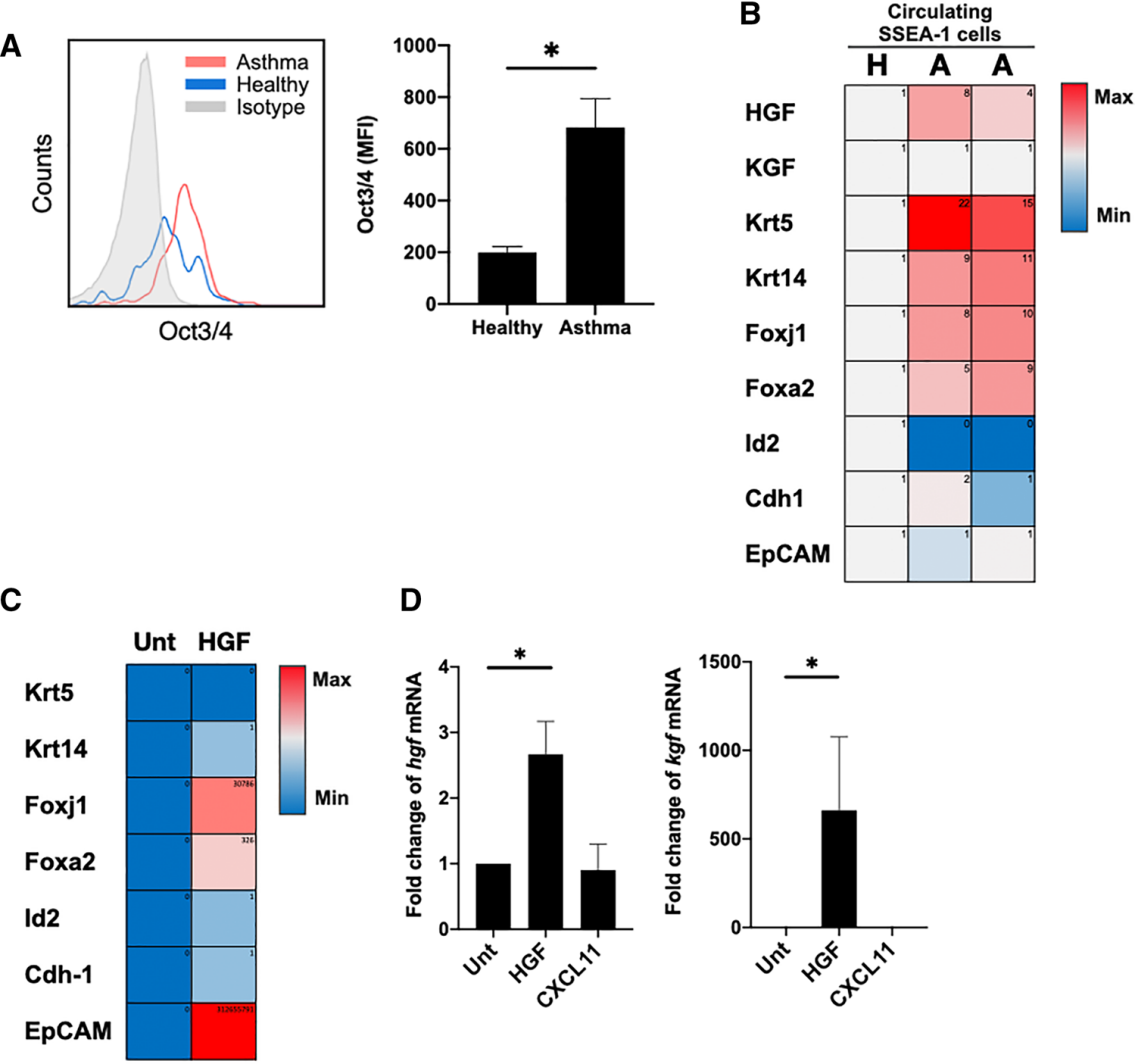
Circulating SSEA-1^+^ cells express tissue repair-associated genes in response to HGF. **A** FACS analysis of Oct3/4 expression in the circulating SSEA-1^+^ cells of asthmatic and healthy mice (*n* = 3/group). At least 300 events/sample were acquired. Data are mean ± SEM. Student's *t* test was performed between healthy and asthma groups. *P < 0.05 shows statistically significant. **B** Heatmap of selected mRNA levels by RT-QPCR in circulating SSEA-1^+^ cells of 1 healthy (abbreviated as “H”) and 2 asthmatic (abbreviated as “A”) mice. *Gapdh* was used as an endogenous control. **C** Heatmap of selected mRNA levels by RT-QPCR in circulating SSEA-1^+^ cells treated with or without HGF. *Gapdh* was used as an endogenous control. Data are representative of two independent experiments. **D** *Fgf* and *hgf* mRNA levels in circulating SSEA-1^+^ cells treated with HGF or CXCL11. *Gapdh* was used as an endogenous control. Data are mean ± SEM. Statistical significance was determined using ANOVA with Tukey’s multiple-comparison testing between all groups. *P < 0.05 shows statistically significant. Data are representative of two independent experiments

To further clarify the biological significance of circulating SSEA-1^+^ cells in lung repair after allergen challenge in asthmatic conditions, we isolated circulating SSEA-1^+^ cells from healthy and asthmatic mice. FACS analysis revealed that Oct3/4 expression of circulating SSEA-1^+^ cells was increased in asthmatic mice (Fig. 4A). RT-QPCR analysis showed that the gene expression of circulating SSEA-1^+^ cells was different between healthy and asthmatic mice. Since hepatocyte growth factor (HGF) and keratinocyte growth factor (KGF) are critical in lung tissue repair [6, 22], the expression of HGF was increased in asthmatic circulating SSEA-1^+^ cells. Moreover, in circulating SSEA-1^+^ cells derived from asthmatic mice, the expression of the inhibitor of differentiation gene *Id2* (inhibitor of DNA binding 2) was inhibited, and that of various lung development genes—*Krt5* for basal cells, *Krt14* for epithelial cells, *Foxj1* (forkhead box j1) for ciliary proteins, and *Foxa2* (forkhead box protein A2) for alveolarization—were enhanced (Fig. 4B).

To further address whether circulating SSEA-1^+^ cells were responsible for lung tissue repair, we isolated circulating SSEA-1^+^ cells and treated them with HGF and KGF. RT-QPCR showed that the expression of *Foxj1*, *EpCAM*, and *Foxa2* was enhanced in HGF-treated circulating SSEA-1^+^ cells (Fig. 4C), but there was no significant change in KGF-treated circulating SSEA-1^+^ cells (data not shown). Moreover, HGF-treated circulating SSEA-1^+^ cells exhibited increased *hgf* and *kgf* expression (Fig. 4D). These data suggested that asthmatic circulating SSEA-1^+^ cells not only endogenously expressed high HGF but also induced more HGF through an autocrine manner and then enhanced lung tissue repair in asthmatic mice.

### Circulating SSEA-1^+^ cells alleviated airway inflammation in asthmatic mice

**Fig. 5:**
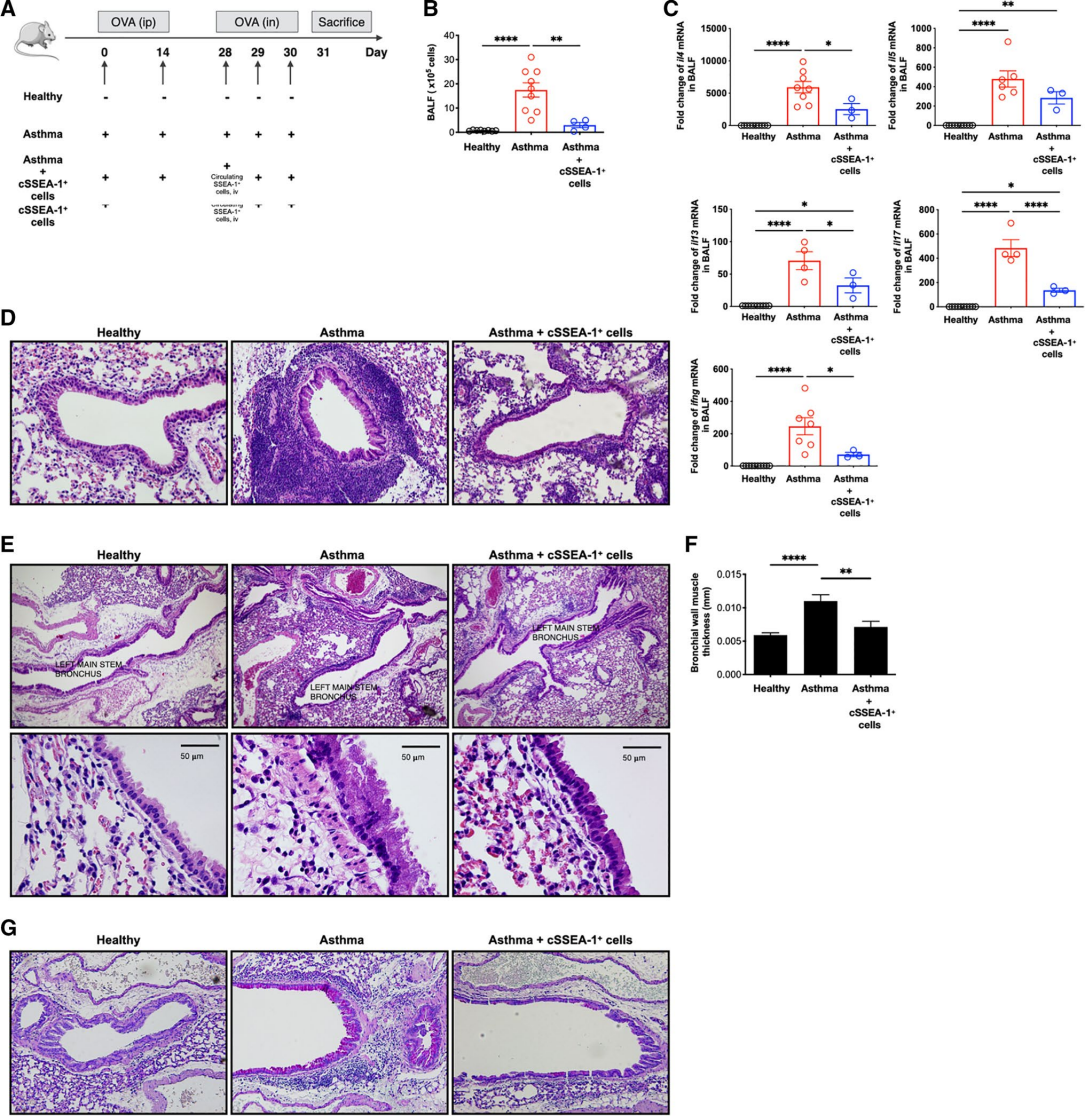
Circulating SSEA-1^+^ cells ameliorated OVA-induced allergic airway inflammation. **A** Flowchart of the method used to produce the OVA-induced airway inflammation model. Enriched circulating SSEA-1^+^ cells were adoptively transferred into asthmatic recipient mice after the first inhaled OVA challenges. Intraperitoneal injection (ip); intravenous injection (iv); intranasal administration (in). **B** BALF was taken 24 h after the last intranasal administration of OVA exposure. Cell number in recovered BALF was counted after staining by trypan blue. Data are mean ± SEM and are representative of three independent experiments. Statistical significance was determined using ANOVA with Tukey’s multiple-comparison testing between all groups. **P < 0.01 and ****P < 0.0001 show statistically significant. **C** RT-QPCR analyses of *il4*, *il5*, *il13*, *ifng*, and *il17* mRNA expression in BALF cells. *Gapdh* was used as an endogenous control. Data are mean ± SEM and are representative of three independent experiments. Statistical significance was determined using ANOVA with Tukey’s multiple-comparisons testing between all groups. *P < 0.05, **P < 0.01, and ****P < 0.0001 show statistically significant. **D** Histological sections of the lungs stained with H&E in circulating SSEA-1^+^ cells treated asthmatic mice (magnification: × 100). Data are representative of three independent experiments. **E** Bronchial mucosal lesions. Upper panel (magnification: × 40) showed selection of tissue section including left main stem bronchus where changes can be measured accurately. Lower panel (magnification: × 400) showed the hypertrophy of submucosal smooth muscle. **F** The quantification of thickness of submucosal muscle. Data are means ± SEM. Statistical significance was determined using ANOVA with Tukey’s multiple-comparison testing between all groups. **P < 0.01 and ****P < 0.0001 show statistically significant. **G** Lung tissues stained with PAS for mucus production (magnification: × 100)

To evaluate the therapeutic potential of circulating SSEA-1^+^ cells in asthmatic model, mice were immunized with OVA and challenged with OVA aerosol for 3 consecutive days. For the treatment group, mice were exposed to OVA inhalation and received circulating SSEA-1^+^ cells simultaneously (Fig. 5A). The total cell counts in bronchoalveolar lavage fluid (BALF) were significantly decreased in the circulating SSEA-1^+^ cell-treated group compared to those in the untreated group (Fig. 5B). RT-QPCR showed that treatment with circulating SSEA-1^+^ cells significantly inhibited *il4*, *il5*, *il13*, *il17,* and *ifng* mRNA expression in BALF cells (Fig. 5C). Notably, histological analysis of lung tissue sections revealed circulating SSEA-1^+^ cells decreased the infiltration of inflammatory cells into peribronchovascular and goblet cell hyperplasia areas (Fig. 5D), the thickened smooth muscle layers (Fig. 5E, F), and the expression of periodic acid schiff (PAS)-positive mucus-containing goblet cells (Fig. 5G) in OVA-induced asthmatic mice.

**Fig. 6:**
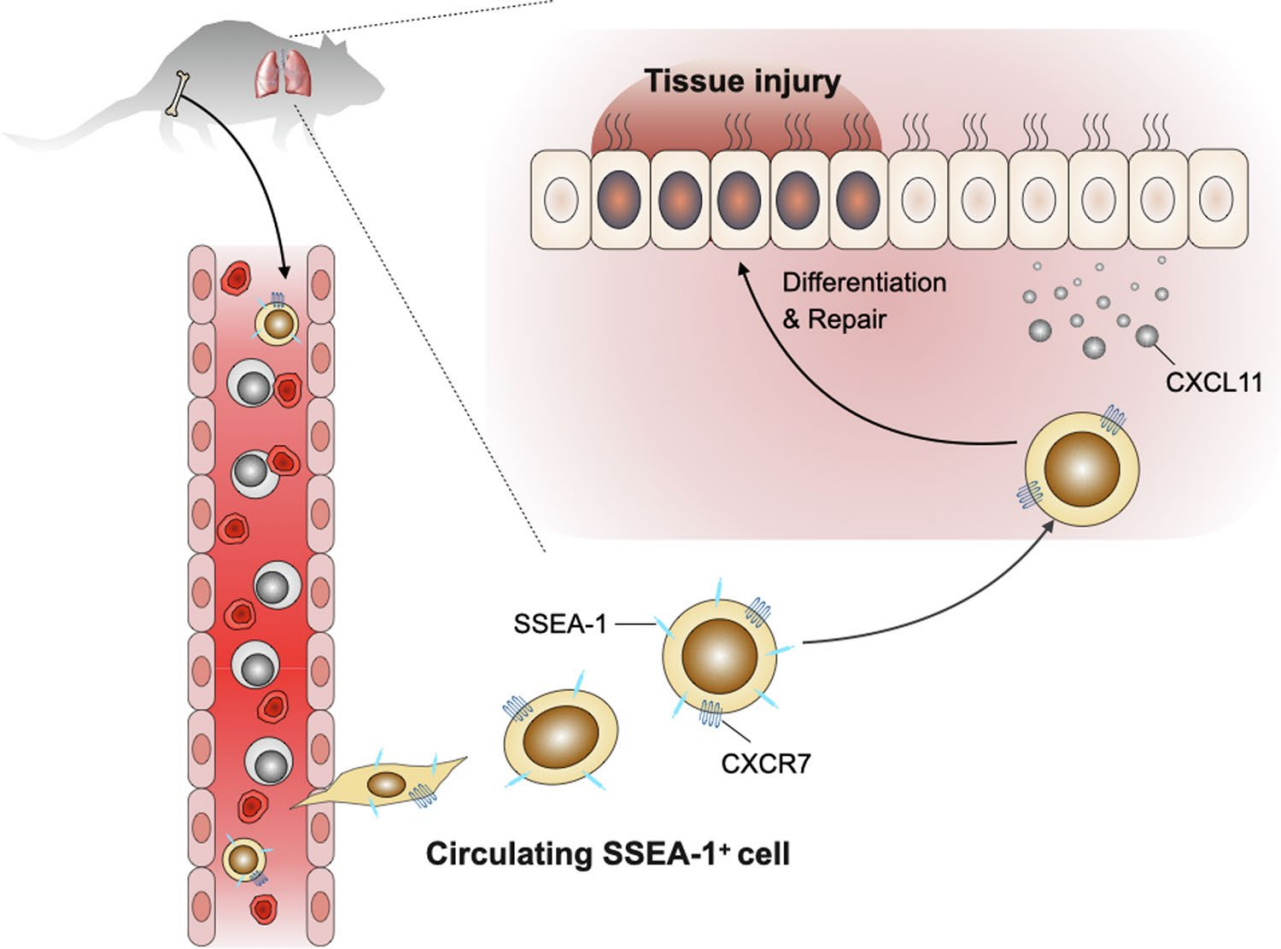
Models for s rescue mechanism of circulating SSEA-1^+^ cells in allergic airway inflammation.

These findings demonstrated that circulating SSEA-1^+^ cells have a beneficial effect on injury repair, repress inflammatory mediators, and might play a pivotal role in the resolution of airway inflammation (Fig. 6).

## Discussion

Neonatal SSEA-1^+^ PSCs have been shown to exert a protective effect to reduce allergen-induced airway inflammation and damage in an asthmatic mouse model [4]. We previously showed that the number of SSEA-1^+^ PSCs was increased in neonatal mice and decreased in adult mice in an age-dependent manner. SSEA-1^+^ PSCs are physiologically rare, but these cells do exist in adult lung tissue [4]. These findings raise the question of why endogenous SSEA-1^+^ PSCs in adult mice cannot exert protective functions after allergen challenge. It is possible that the number of endogenous SSEA-1^+^ PSCs in adult mice is too low to protect against airway inflammation or that the function of SSEA-1^+^ PSCs in adult mice is different from that of these cells in neonatal mice. In the sphere-forming assay, we found that the sphere-forming efficiency was lower for adult SSEA-1^+^ cells than for neonatal SSEA-1^+^ PSCs. They also failed even when seeding a threefold higher cell number to enhance the sphere-forming efficiency in adult SSEA-1^+^ cells. Moreover, our recent study [17] further investigated and characterized the difference between adult and neonatal lung SSEA-1 cells. No significant difference in the viability and phenotype changes of pulmonary SSEA-1^+^ cells derived from adult and neonatal mice. However, the sphere-forming ability and the bio-functions of SSEA-1^+^ cells derived from adult and neonatal mice are different. The pluripotent and differentiation ability in adult lung SSEA-1 cells were limited. These data suggested that the function of SSEA-1^+^ cells in adult mice is different from that of cells derived from neonatal mice. Adult SSEA-1^+^ cells might need other stimuli, such as growth factors or alarmin signals, to initiate their expansion and differentiation ability.

In this study, we found that lung SSEA-1^+^ PSCs were almost depleted; however, a unique population of SSEA-1^+^ cells was enriched in the peripheral blood of OVA- and HDM-induced asthmatic mice. Circulating SSEA-1^+^ cells were significantly enriched and specifically homed to the lung through the CXCR7–CXCL11 axis in response to inhaled antigen in asthmatic mice. Adoptive transfer of circulating SSEA-1^+^ cells into asthmatic mice alleviated airway inflammation. Therefore, we have identified a critical role of circulating SSEA-1^+^ cells in an asthmatic attack.

The airway epithelium in asthma is susceptible to environmental injury and responds by remodeling secondary to inflammation, epithelial activation, and epithelial damage [13]. Murine studies using asthmatic models have shown that after allergen inhalation, fibrocytes localize to the airway mucosa, where they acquire a myofibroblast phenotype [28]. The levels of circulating CD34^+^ fibrocytes are higher in asthmatic patients with chronic airflow obstruction than in those without chronic airflow obstruction [34]. Circulating CD34^+^ fibrocytes express CD45, collagen I, and α-smooth muscle actin, and have been suggested to be precursors of bronchial myofibroblasts in asthma [8, 28]. Circulating SSEA-1^+^ cells differ from circulating CD34^+^ fibrocytes, because circulating SSEA-1^+^ cells have been defined as CD45-negative cells in peripheral blood.

Inflammation is a nonspecific biological response of tissues to harmful stimuli. Excess or prolonged inflammatory responses resulted in a wide variety of acute and chronic diseases in various organs including the lungs [38]. In GFP/BMT asthmatic model, circulating SSEA-1^+^ cells migrated to lung tissue and transdifferentiated into alveolar space when mice were exposed to HDM 1 month later. These data indicated that circulating SSEA-1^+^ cells were beneficial for tissue repair and regeneration, and thus, circulating SSEA-1^+^ cells might help to initiate the resolution of the inflammation. The circulating SSEA-1^+^ cells were increased in the short-term asthmatic model, but the cell number might not be sufficient to initiate the resolution of inflammation promptly. In contrast, chronic allergic airway inflammation results in irreversible fibrosis and lung structural change. Meanwhile, the increased circulating SSEA-1^+^ cells in chronic asthmatic mice might not help to reverse this kind of pathological change. It will be interesting to investigate which stimuli such as cytokines or inflammatory mediators for initiation of the circulating SSEA-1^+^ cells, and how to induce or amplify these cells to suspend unwanted reactions.

CXCR7, also known as atypical chemokine receptor 3 (ACKR3), is not a classic GPCR and signals primarily through β-arrestin recruitment and therefore belongs to the ACKR class [24]. It has been reported that the pulmonary epithelium highly expresses CXCR7 to promote transepithelial polymorphonuclear neutrophil (PMN) migration from the lung interstitium into the BALF [20, 23]. Inhibition of CXCR7 have been shown to increase tight junction formation by downregulating CXCL12 and second chemokine receptor CXCR4 expression [20]. CXCR7 regulates progenitor cell homing to tissues [19], since CXCR7 is involved in embryonic development [30], directional cell migration [7], and immune functions [21]. Although inhibition of CXCR7-CXCL12 signaling seems to maintain the pulmonary epithelium barrier, study of the CXCR7–CXCL11 axis related to airway inflammation is limited. The present study showed that circulating SSEA-1^+^ cells migrated to lung tissue through CXCR7–CXCL11 signaling; we speculated that targeting CXCR7 might affect the initiation of repair and rescue systems by circulating SSEA-1^+^ cells. Moreover, we cannot rule out the possibility that circulating SSEA-1^+^ cells express CXCR7 and compete for/exhaust binding of CXCL12, thereby decreasing airway inflammation and maintaining the epithelial barrier in asthma.

The process of cellular differentiation is dynamic and under strict regulation by transcription factors. Our results showed that transferred circulating SSEA-1^+^ cells migrated to the lung and lost SSEA-1 expression in response to allergen inhalation. We suggested that asthmatic mice-derived circulating SSEA-1^+^ cells might initiate differentiation and response to lung repair and loss of SSEA-1 expression. This efficient migration to the inflamed lung tissue occurred through the CXCR7–CXCL11 axis. However, gene expression did not change in CXCL11-treated circulating SSEA-1^+^ cells. CXCL11 provides a chemoattractant signal for circulating SSEA-1^+^ cell migration from peripheral blood into lung tissue, but might not affect the differentiation of circulating SSEA-1^+^ cells. It has previously been shown that CXCL11 concentrations in nasal lavages in allergic patients are increased after nasal allergen challenge [33]. Not only is the frequency of circulating SSEA-1^+^ cells increased in asthmatic mice, but the gene expression pattern is different between healthy and asthmatic mice.

HGF is a multifunctional cytokine, which prevents fibrotic remodeling and induces cellular motility, survival, proliferation, and morphogenesis [22]. In asthmatic mice, HGF attenuated AHR and allergen-induced airway remodeling and inflammation [14]. Previous studies reported that mouse lung epithelial cell adhesion molecule (EpCAM)-positive epithelial stem cells could differentiate into alveolar cells [16]. EpCAM-positive epithelial cells from mouse lungs could culture them as organoids to maintain epithelial stem cell properties [29]. In our in vitro data, we found that HGF upregulated *EpCAM*, *Foxj1*, and *Foxa2* expression in circulating SSEA-1^+^ cells isolated from asthmatic mice but not from healthy mice. Foxj1 is a critical factor for cilia formation [39]. FOXA2 plays crucial roles in regulating embryonic lung development and postnatal lung homeostasis [5]. All these pieces of data suggested that when circulating SSEA-1^+^ cells migrated to the inflamed lung tissue through the CXCR7–CXCL11 axis, HGF can further stimulated EpCAM expression to induce pluripotent properties in these cells and then repaired and redifferentiated the bronchiolar epithelium after lung injury through upregulating Foxj1 and Foxa2. Although we showed that HGF treatment enhances differentiation gene expression in circulating SSEA-1^+^ cells, which external triggers contribute to the induction and differentiation of circulating SSEA-1^+^ cells remain to be further defined.

Our data showed a 700-fold increase in CCR10 mRNA expression in circulating SSEA-1^+^ cells at 3 days post-challenge, while the mRNA expression of the genes encoding two cognate ligands, CCL27 and CCL28, was not significantly different in asthmatic and healthy mouse-derived lung tissues. This result is consistent with the finding that CCL28 mRNA can be detected within normal and asthmatic lungs [35]. Although there is no evidence indicating increased CCL28 expression in asthmatic patient-derived lung tissue, a previous study reported that serum CCL28 levels in children with bronchial asthma are significantly higher than those in controls [9]. Therefore, this finding raises the possibility that CCL28 might stimulate the migration of circulating SSEA-1^+^ cells highly expressing CCR10 from the BM reservoir into the circulation, which then migrate into inflamed lung tissue via the CXCL11–CXCR7 axis.

Our study highlights a previously undescribed cell population in peripheral blood-circulating SSEA-1^+^ cells derived from BM. This cell population was enriched after allergen challenge. We conclude that reinforcement of circulating SSEA-1^+^ cells from peripheral blood to lung tissue might be a rescue mechanism for acute inflammatory responses after allergen challenge in asthmatic conditions. Understanding their mode of action should facilitate attempts to harness them therapeutically for either cell therapy or small-molecule approaches.

## Supplementary Information

Below is the link to the electronic supplementary material.

Supplementary file1 (PDF 3323 kb)

## Data Availability

The data that support the findings of this study are available within the supplementary information section.

## Abbreviations

ABCA3: ATP-binding cassette, class A3
ACKR3: Atypical chemokine receptor 3
AHR: Airway hyperresponsiveness
BADJ: Bronchiole-alveolar duct junction
BALF: Bronchoalveolar lavage fluid
BM: Bone marrow
BMT: Bone marrow transplantation
CCSP: Clara cell secretory protein
CFSE: Carboxyfluorescein succinimidyl ester
ELISA: Enzyme-linked immunosorbent assay
EpCAM: Epithelial cell adhesion molecule
FACS: Fluorescence-activated cell sorting
Foxa2: Forkhead box protein A2
Foxj1: Forkhead box j1
FVS780: Fixable Viability Stain 780
HDM: House dust mite
HGF: Hepatocyte growth factor
Id2: Inhibitor of DNA binding 2
IL: Interleukin
KGF: Keratinocyte growth factor
Krt: Keratin
MSC: Mesenchymal stem cell
MTT: 3-(4,5-Dimethylthiazol-2-yl)-2,5-diphenyltetrazolium bromide
NLAC: National Laboratory Animal Center
OVA: Ovalbumin
PAS: Periodic acid schiff
p63: Tumor protein p63
PSC: Pulmonary stem/progenitor cells
RT-QPCR: Reverse transcription quantitative real-time PCR
T1α: Podoplanin
TSLP: Thymic stromal lymphopoietin
