# Supplementary material for: Circulating SSEA-1+ stem cell-mediated tissue repair in allergic airway inflammation
Source: Cell Mol Life Sci. 2022 Jun 7;79(7):347. doi: 10.1007/s00018-022-04366-3 (PMC9174110; doi:10.1007/s00018-022-04366-3)
Supplement: Supplementary file 1 — Supplementary file1 (PDF 3323 kb) [file 18_2022_4366_MOESM1_ESM.pdf]

Supplementary Information

Circulating SSEA-1$^+$ Stem Cell-Mediated Tissue Repair in Allergic Airway Inflammation

Chiao-Juno Chiu, Chien-Chia Liao, Yu-Hsiang Hsu, and Bor-Luen Chiang

MATERIALS AND METHODS

Animals

BALB/c, C57BL/6 and C57BL/6-Tg(Actb-flEmGFP-FusionRed)10Sji/Narl male mice (6–8 weeks old) were purchased from the National Laboratory Animal Center (Taiwan) and maintained in the Animal Center of the College of Medicine, National Taiwan University. Animal care and handling protocols were approved by the Animal Welfare Committee of National Taiwan University. BALB/c male mice were used in all *in vivo* and *in vitro* studies except HDM-induced asthmatic model and BMT experiments.

HDM-induced asthmatic animal model

The animal model of HDM-induced asthmatic mice was established according to and modified from the protocol of the laboratory of Bart N Lambrecht (Plantinga et al. 2013). Briefly, BALB/c and C57BL/6 male mice were sensitized to 1 μg of HDM (Stallergenes Greer) in 35 μl of PBS via intranasal (i.n.) administration. After 7 days, the mice were injected with 10 μg of HDM (i.n.) daily for 5 consecutive days. The mice were sacrificed 1 day after the last HDM exposure. Peripheral blood-derived SSEA-1^{+} cells were analysed on the indicated days.

OVA/alum-induced asthmatic animal model

BALB/c mice were sensitized to OVA (Sigma-Aldrich) via intraperitoneal (i.p.) injection of

100 µg of OVA with 4 mg of aluminium hydroxide (Pierce Chemical) on day 0. On days 14 and 28, the mice were boosted with 50 µg of OVA and 4 mg of aluminium hydroxide. On days 42–44, the mice were challenged for 30 min with an aerosol of 1% OVA in PBS delivered from a jet nebulizer. For the chronic asthma model, mice were challenged for 3 consecutive days every week for 3 months. Then, the mice were sacrificed, and samples were collected for further analysis.

Bone marrow transplantation (BMT)

C57BL/6 male mice (7 weeks of age) were irradiated with 9.5 Gy (Rad Source, RS2000). One day prior to transplantation, mice received 1×10^7 BM cells from C57BL/6-Tg(Actb-flEmGFP-FusionRed)10Sji/Narl male mice (GFP-reporter mice; for the experiment of cellular source of circulating SSEA-1$^+$ cells) or 5×10^6 BM from C57BL/6 mice along with 1×10^5 circulating SSEA-1$^+$ cells from GFP-reporter mice (for the experiment of transdifferentiaion). A minimum of 1 month after transplantation, donor chimaerism of total nucleated cells in the blood of control chimaeras was confirmed by flow cytometric analysis using GFP markers.

Preparation of pulmonary single-cell suspensions

Lung tissues were washed with PBS, cut into small pieces and then digested in MEM (Sigma-Aldrich) in the presence of 1 mg/ml protease-type XIV (Sigma-Aldrich) and 0.5 mg/ml

DNase I (Invitrogen) for 18 hours at 4°C. PBS containing 5% foetal bovine serum (HyClone) was added to neutralize protease activity, and pipetting was performed to obtain a pulmonary single-cell suspension. The cell extract was filtered through a 100 μm nylon mesh (BD Biosciences) to remove debris. The single-cell was washed and resuspended in FACS buffer.

Identification and quantitation of murine circulating SSEA-1^{+} cells

Heparinized whole blood (100 μl) was pretreated with FcR-blocking reagent (BD Bioscience) to block nonspecific antibody binding and stained with specific monoclonal antibodies on ice for 10 min (all antibodies used in this study were listed in Supplementary Information Tab. 1). Isotype-matched control antibodies were used as negative controls. Then, red blood cells were lysed with lysis solution (BD Bioscience) and washed twice with FACS buffer. Samples were washed and analyzed on a FACSCanto II with FACSDiva software (BD Bioscience).

Enrichment of SSEA-1^{+} cells from peripheral blood and pulmonary single-cell suspension

Red blood cells in whole blood were lysed with lysis solution (BD Bioscience) and washed twice with PBS containing 2% FBS. FcR-blocking reagent (BD Bioscience) was added and incubated for 10 min to block nonspecific antibody binding. Thereafter, cells were incubated with anti-SSEA-1 microbeads (Miltenyi Biotec), and SSEA-1^{+} cells were enriched by using a

magnetic separator. The purity of the SSEA-1^+ cells was greater than 90%, as determined by FACS analysis. For *in vivo* adoptive transfer experiment, 1×10^4 circulating SSEA-1^+ cells were intravenously injected in to a mouse.

CFSE-labelling and tracking the migration *in vivo*

Enriched 1×10^5 circulating SSEA-1^+ cells were resuspended in PBS containing 2 μM CFSE (BD Bioscience) at 37°C for 10 min. Then wash twice with PBS containing 5% foetal bovine serum (FBS; Invitrogen). 1×10^4 CFSE-labeled circulating SSEA-1^+ cells were intravenous injected into each mouse. To verify the transferred circulating SSEA-1^+ cells in various organs, tissues were washed with PBS, cut into small pieces and then digested in PBS in the presence of 2.5 mg/ml collagenase II (Sigma-Aldrich) and 0.5 mg/ml DNase I (Invitrogen) for 40 min at 37°C. The test tubes were gently inverted and shaken every 5–10 min. Then, PBS containing 5% FBS was added to neutralize the enzyme activity, and pipetting was performed to obtain a pulmonary single-cell suspension. The cell extract was filtered through a 100 μm nylon mesh (BD Biosciences) to remove debris. The cell suspension was washed and resuspended in FACS buffer, and these cells were then ready FACS analysis. For the SSEA-1 retention experiment, single-cell suspension from each organ was stained with the same clone of anti-SSEA-1 antibody conjugated with PerCP/Cy5.5 and analyzed by cytometry.

Cell culture and immunofluorescence staining of spheroids

For spheroid culture, 1–2×10^5 lung SSEA-1$^+$ cells were isolated and suspended in 100 µl of 50% growth factor-reduced Matrigel (BD Biosciences) in MCDB201 medium (Sigma-Aldrich) and placed in a 24-well Transwell insert with a 0.4 µm pore size (Falcon). Cell-containing Matrigel was allowed to solidify for 15 min at 37°C. MCDB201 medium supplemented with 5% FBS, insulin-transferrin-selenium (Gibco), 100 U/ml penicillin, 100 µg/ml streptomycin and 25 ng/ml epidermal growth factor (BD Biosciences) was added to the lower chamber. The Rock inhibitor Y27632 (Selleckchem) was included in the medium during the first two days, and the medium was replaced every two days. After 14–21 days, the primary spheres were collected and fixed with Bouin's solution (Sigma-Aldrich) for 10 min at room temperature or fixed with 100% methanol for 10 min at -20°C. The nonspecific binding of sections was blocked by 3% BSA for 1 hour at room temperature. Samples were incubated with primary antibodies (Supplementary Information Tab. 1) overnight at 4°C. After washing, Alexa Fluor-coupled secondary antibodies (Invitrogen) were used to detect the bound primary antibodies for 1 hour at room temperature. Confocal microscopy (ZEISS, LSM 510 META and LSM780) was performed to visualize the stained cells.

Viability of cell suspension from spheroids

After 14 days of cultivation, the Matrigel in the spheroids were digested by incubation in accumax solution (Innovative cell technologies) at room temperature for 2 hr. The spheres were harvested and digested with 0.05% trypsin/EDTA (Corning) solution for 3 min at 37 ˚C, followed by inactivation of trypsin by FBS. The cells were washed twice and resuspended in PBS then passed through a 40-um cell strainer. Cell viability was verified by stained with BD Horizon™ Fixable Viability Stain 780 (FVS 780; BD Biosciences) according to the manufacturer's protocol. Flow cytometry analysis was then performed to quantify viable or dead cells.

Real-time quantitative-polymerase chain reaction (RT-QPCR)

Total RNA was extracted using TRIzol reagent (Invitrogen) according to the manufacturer's instructions. First-strand cDNA was synthesized with random hexamers using Moloney murine leukaemia virus (MMLV) reverse transcriptase (Clontech). RT-QPCR was performed using SYBR Green for quantitative PCR (Applied Biosystems) with gene-specific primers (Supplementary Information Tab. 2) and analysed by using the 7500 FAST Real-Time PCR System (Applied Biosystems). *Gapdh* was used as an endogenous control. The results were normalized to *Gapdh* or β-actin expression and analysed using the ΔΔCt method. The relative multiples of changes in mRNA expression were determined by calculating $2^{-\Delta\Delta Ct}$.

CXCL11 ELISA

The levels of CXCL11 in left lung lobe homogenates were analyzed using Duoset ELISA kits (R&D Systems), following the manufacturer's instructions.

Histology and immunohistochemistry (IHC)

Lung tissues were dissected and fixed in 4% paraformaldehyde in PBS and processed into serial paraffin sections using standard procedures. In brief, the sections were blocked with antibody diluent (Dako) for 1 hour at room temperature. The sections were then incubated with primary antibodies at 4°C overnight. Antibodies against SSEA-1 were used (1:50), and isotype-matched control antibodies were used as a negative control. Confocal microscopy (ZEISS, LSM 510 META and LSM780) was performed to visualize the stained cells.

Migration assay

Freshly enriched circulating SSEA-1^+ cells (3000 cells in 200 µl/well) were loaded into Transwell inserts with a 5 µm pore size (Falcon) and preincubated for 30 min in migration medium (MCDB201 medium supplemented with 2% FBS and antibiotics). Thereafter, 600 µl of fresh migration medium containing 0, 100, or 300 ng/ml CXCL11 (Biolegend) was added to the lower compartment of the Transwell. For CXCL11 neutralization experiment, 300 ng/ml CXCL11 was preincubated with 3 µg/ml anti-CXCL11 antibody (Biolegend) or isotype-

matched antibody for 30 min on ice. After 4 hours of incubation at 37°C and with 5% CO_2, migrated circulating SSEA-1$^+$ cells in the lower compartment were collected. The absolute cell number was analysed by using a FACSCanto II with FACSDiva software (BD Bioscience). CountBright™ absolute counting beads (Thermo Fisher, UK) were used as a volumetric standard.

Statistical analysis

All statistical analyses were performed with Prism 9.0 (GraphPad Software, San Diego, CA) software. Two-group comparisons were made with Student's *t*-test. Groups of three or more were analysed by ANOVA with a post hoc Tukey honestly significant difference (HSD) test. P values < 0.05 were considered significant.

Supplementary Information Table 1. Specification of antibodies.

Antibody	Conjugate	Clone	Source
CD9	PE	MZ3	Biolegend
CD24	PE	M1/69	Biolegend
CD26	FITC	H194-112	BD Bioscience
CD29	PE	HMβ1-1	Biolegend
CD31	PE-Cy7	MEC 13.3	BD Bioscience
CD44	PE	IM7	BD Bioscience
CD45	PerCP	30-F11	BD Bioscience
CD47	FITC	miap301	BD Bioscience
CD54	PE	YN1/1.7.4	Biolegend
CD63	PE	NVG-2	BD Bioscience
CD73	PE	eBioTY/11.8	eBioscience
CD98	PE	RL388	BD Bioscience
CD105	PE	MJ7/18	BD Bioscience
CD326	APC	G8.8	BD Bioscience
CXCR7	PE	8F11-M16	Biolegend
Oct3/4	Alexa Flour 647	40/Oct-3	BD Bioscience
Sca-1	PE	E13-161.7	BD Bioscience
SSEA-1	Alexa Flour 488	MC480	BD Bioscience
SSEA-1	BV421	MC480	BD Bioscience
SSEA-1	PerCP-Cy5.5	MC480	BD Bioscience
T1α	PE	8.1.1	BD Bioscience
ABCA3	-	3C9	Abcam
Acetylated α-tubulin	-	6-11B-1	Sigma-Aldrich
CCSP	-	EPR19846	Abcam
GFP	-	3H9	Abcam
Krt5	-	EP1601Y	Abcam
P63	-	4A4	Abcam
SPC	-	polyclone	Sigma-Aldrich
SPC	-	EPR19839	Abcam

Supplementary Information Table 2. List of gene-specific primer sets for SYBR Green RTQ-PCR.

Gene symbol	Forward primer	Reverse primer
GAPDH	GATGGGTGTGAACCACGAGA	AGATCCACGACGGACACAT
CCR1	GCGATGCTGAATGTGACAGAAGC	GGCATCCATTTGAGAAGCCTGTC
CCR2	GCTGTGTTTGCCTCTCTACCAG	CAAGTAGAGGCAGGATCAGGCT
CCR3	CCACTGTACTCCCTGGTGTTCA	GGACAGTGAAGAGAAAGAGCAGG
CCR4	GGACTAGGTCTGTGCAAGATCG	TGCCTTCAAGGAGAATACCGCG
CCR5	GTCTACTTTCTCTTCTGGACTCC	CCAAGAGTCTCTGTTGCCTGCA
CCR6	ACAGAGCCATCCGAGTCGTGAT	CTGGTGTAGGCGAGGACTTTCT
CCR7	AGAGGCTCAAGACCATGACGGA	TCCAGGACTTGGCTTCGCTGTA
CCR8	CTGCGATGTGTAAGGTGGTCTC	CCTCACCTTGATGGCATAGACAG
CCR9	GCCATGTTCATCTCCAACTGCAC	CCTTCGGAATCTCTCGCCAACA
CCR10	CAGTCTTCGTGTGGCTGTTGTC	TCACAGTCTGCGTGAGGCTTTC
CCR11	CTCACGACTACAGCCAGTACGA	ATCGCCACAACTACGGAGTTCC
D6	GGCACTGCTATGCGGATTTTGG	AACGCAACCGATGCGGGAGTAA
XCR1	AGAGACACCGAACAGTCAGGCT	TGTCCAGTTGCTGAAGGCTCTC
CX3CR1	GAGCATCACTGACATCTACCTCC	AGAAGGCAGTCGTGAGCTTGCA
CXCR2	CTCTATTCTGCCAGATGCTGTCC	ACAAGGCTCAGCAGAGTCACCA
CXCR3	TACGATCAGCGCCTCAATGCCA	AGCAGGAAACCAGCCACTAGCT
CXCR4	GACTGGCATAGTCGGCAATGGA	CAAAGAGGAGGTCAGCCACTGA
CXCR5	ATCGTCCATGCTGTTCACGCCT	CAACCTTGGCAAAGAGGAGTTCC
CXCR6	GGTTCTTCCTGCCATTGCTCAC	GCAGGAACACAGCCACTACAAG
CXCR7	GACCGCTATCTCTCCATCACCT	GTTGGAAGCAGATGTGACCGTC
DARC	GCGATGCTGAATGTGACAGAAGC	GGCATCCATTTGAGAAGCCTGTC
Hgf	GTCCTGAAGGCTCAGACTTGGT	CCAGCCGTAAATACTGCAAGTGG
Fgf7	TGTTCTGTCGCACCCAGTGGTA	TTCCAACTGCCACGGTCCTGAT
CCL25	AAGGCTAGTCCACTGGAAGAGC	GTGGCACTCCTCACGCTTGTAC
CCL27	CTGCTGAGGAGGATTGTCCAC	CACGACAGCCTGGAGGTGA
CCL28	CAGGGCTCACACTCATGGCT	CCATGGGAAGTATGGCTTCTG
CXCL11	CCGAGTAACGGCTGCGACAAAG	CCTGCATTATGAGGCGAGCTTG

CXCL12	GGAGGATAGATGTGCTCTGGAAC	AGTGAGGATGGAGACCGTGGTG
Krt5	ACCTTCGAAACACCAAGCAC	TTGGCACACTGCTTCTTGAC
Krt14	CAAGGATGCTGAGGAATGGT	CCGGAGCTCAGAAATCTCAC
Foxj1	ACACGTGAAGCCACCCTACT	TGTTCAAGGACAGGTTGTGG
Foxa2	GGGAGCCGTGAAGATGGAA	CCGCGGACATGCTCATG
Id2	TCCTGTCCTTGCAGGCATCTGAAT	AACGTGTTCTCCTGGTGAAATGGC
Cdh1	ACTGTGAAGGGACGGTCAAC	GGAGCAGCAGGATCAGAATC
EpCAM	AACACAAGACGACGTGGACA	GCTCTCCGTTCACTCTCAGG

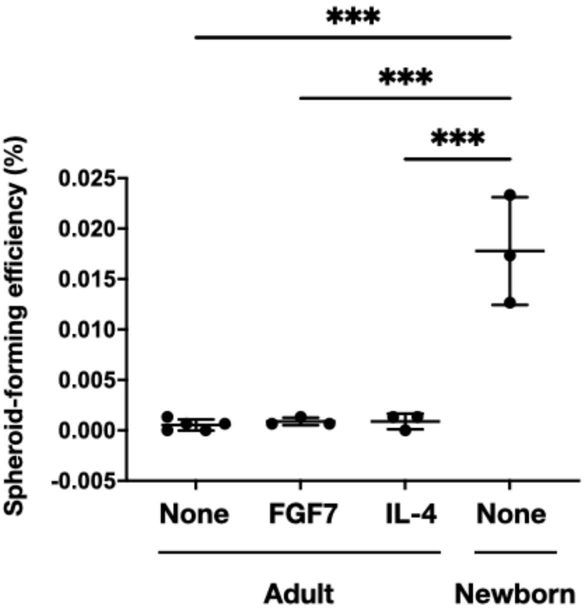

Supplementary Information Figure 1. Lower sphere-forming efficiency of adult pulmonary SSEA-1$^+$ cells in comparison with neonatal SSEA-1$^+$ PSCs. 1.5×10^5 cells of enriched SSEA-1$^+$ cells were suspended in Matrigel in the presence of 25 ng/ml FGF7 and 20 ng/ml IL-4 for 2 weeks. Statistical significance was determined using ANOVA with Tukey's multiple-comparisons testing between all groups. ** *P* <0.01 shows statistically significant.

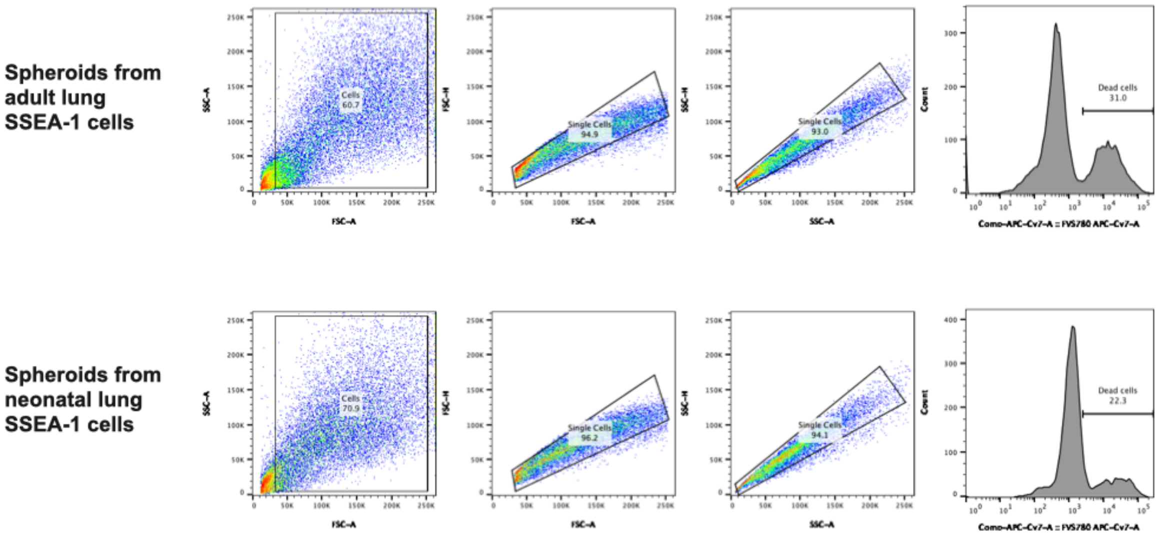

Supplementary Information Figure 2. Adult pulmonary SSEA-1^{+} cells were alive in Matrigel for 14 days cultivation. The viability of pulmonary SSEA-1^{+} cells in spheroids was verified by FVS780 staining and analyzed by FACS.

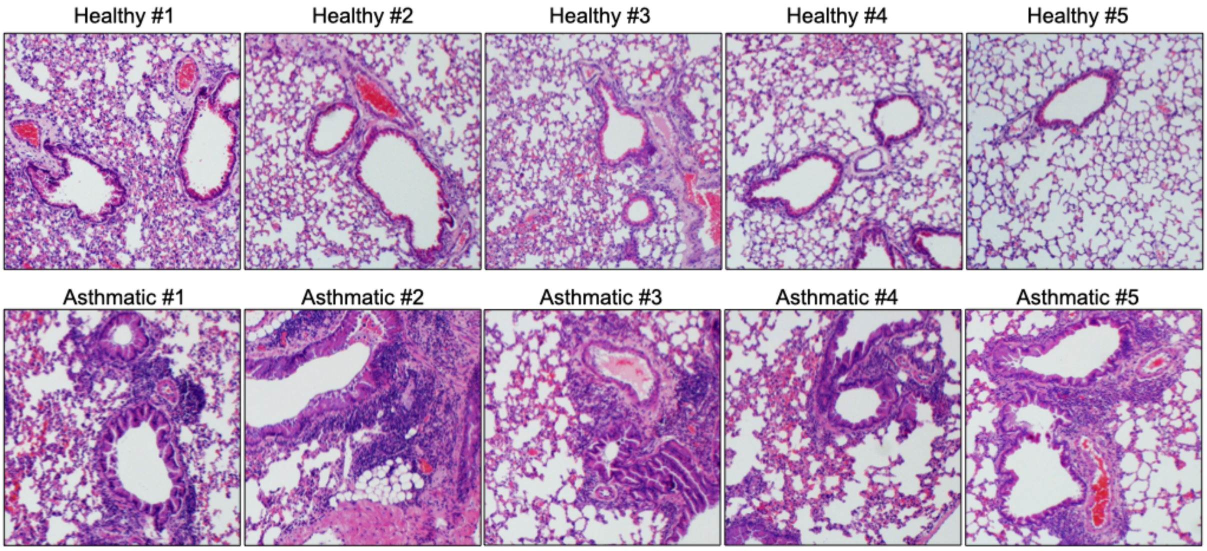

Supplementary Information Figure 3. H&E staining of lung sections in healthy and asthmatic mice (magnification: ×100).

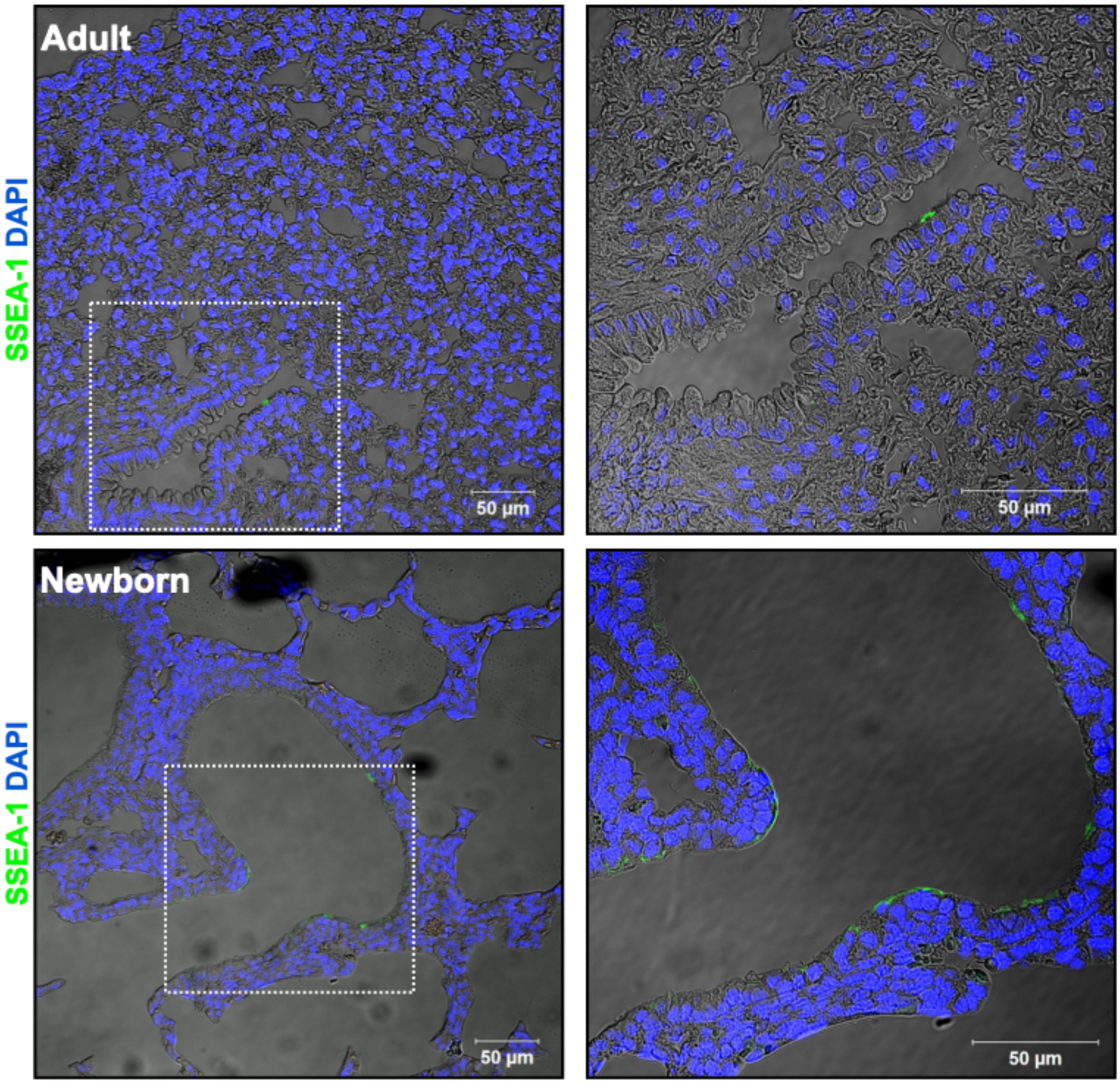

Supplementary Information Figure 4. Distribution of pulmonary SSEA-1$^+$ cells in adult and newborn mice. Lung sections from neonatal (lower) and adult (upper) mice were stained with anti-SSEA-1 (green); the nuclei were counterstained with DAPI (blue).

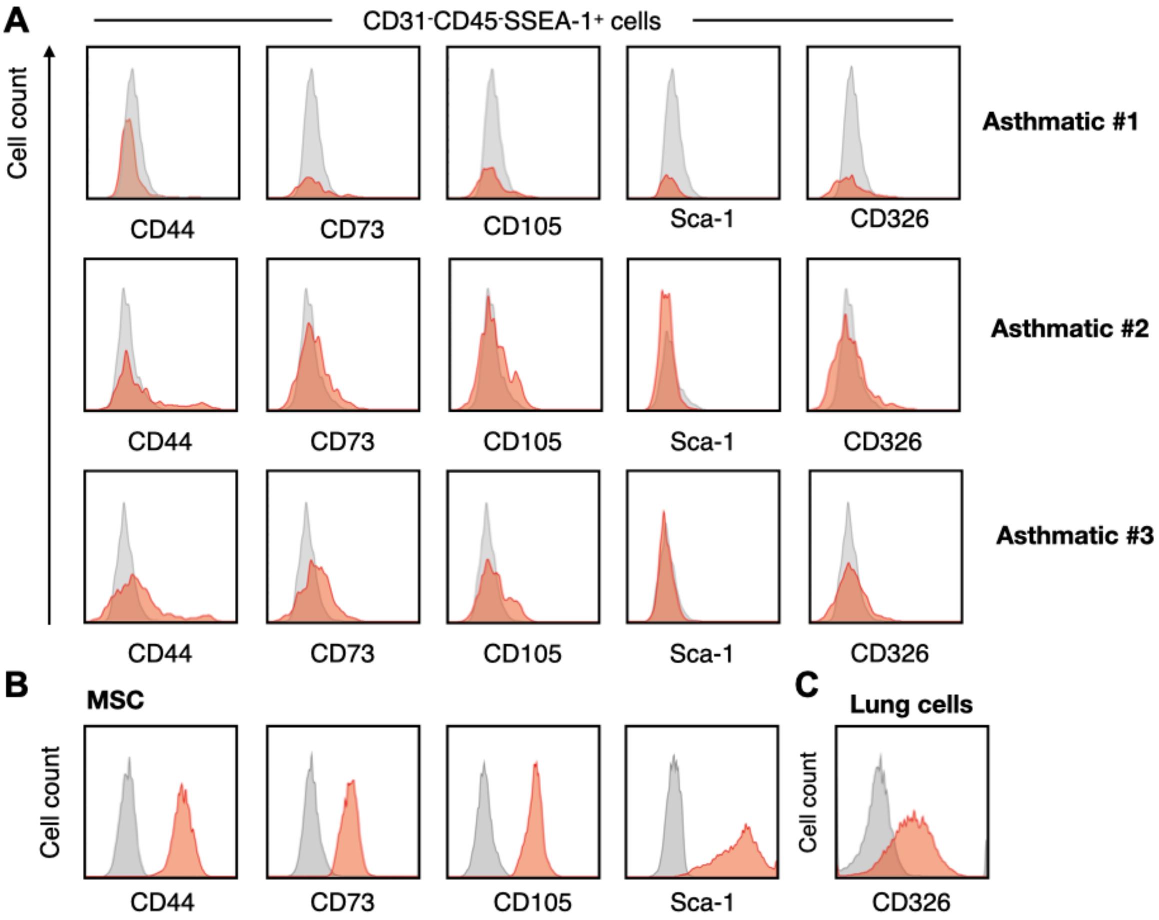

Supplementary Information Figure 5. Characteristics of circulating SSEA-1$^+$ cells. Flow cytometric analysis of surface marker expression in (A) circulating SSEA-1$^+$ cells derived from asthmatic mice, (B) bone marrow-derived MSC, and (C) primary lung epithelial cells. Gray areas represent matched isotype controls. Data are representative of two independent experiments.

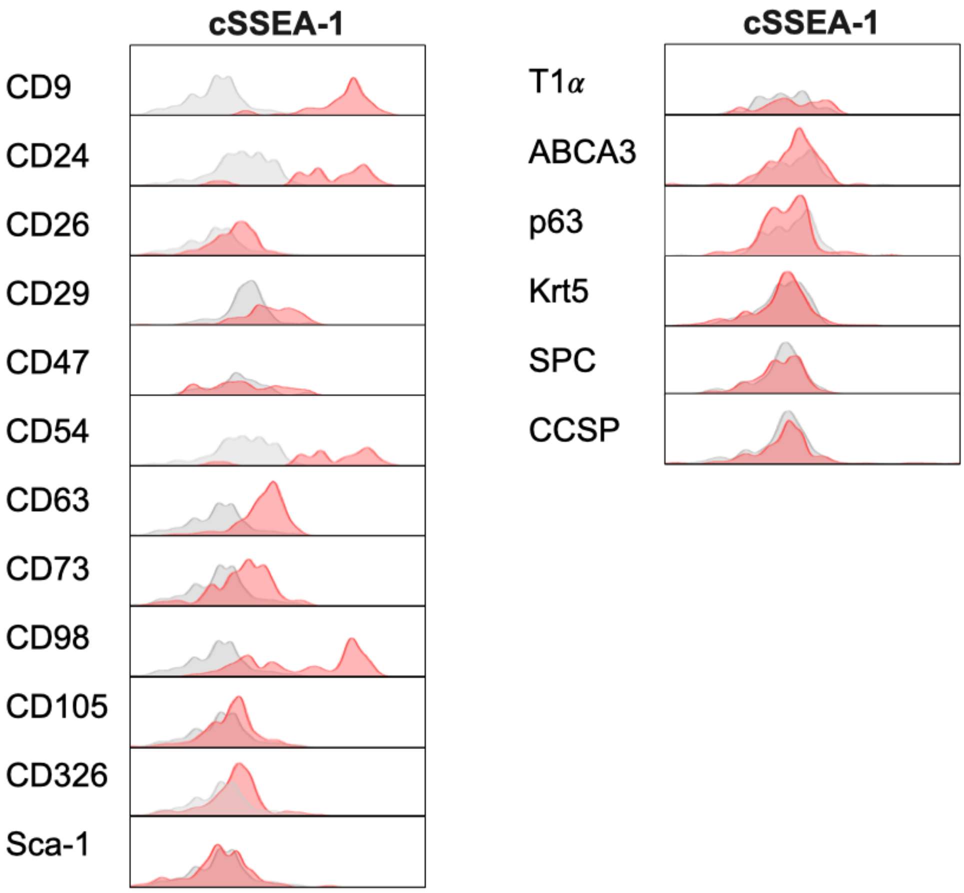

Supplementary Information Figure 6. Surface marker expression of circulating SSEA-1$^+$ cells. Surface marker expression of circulating SSEA-1$^+$ cells was determined by FACS analysis. Gray areas represent matched isotype controls. At least 500 circulating SSEA-1$^+$ cells/sample were acquired.

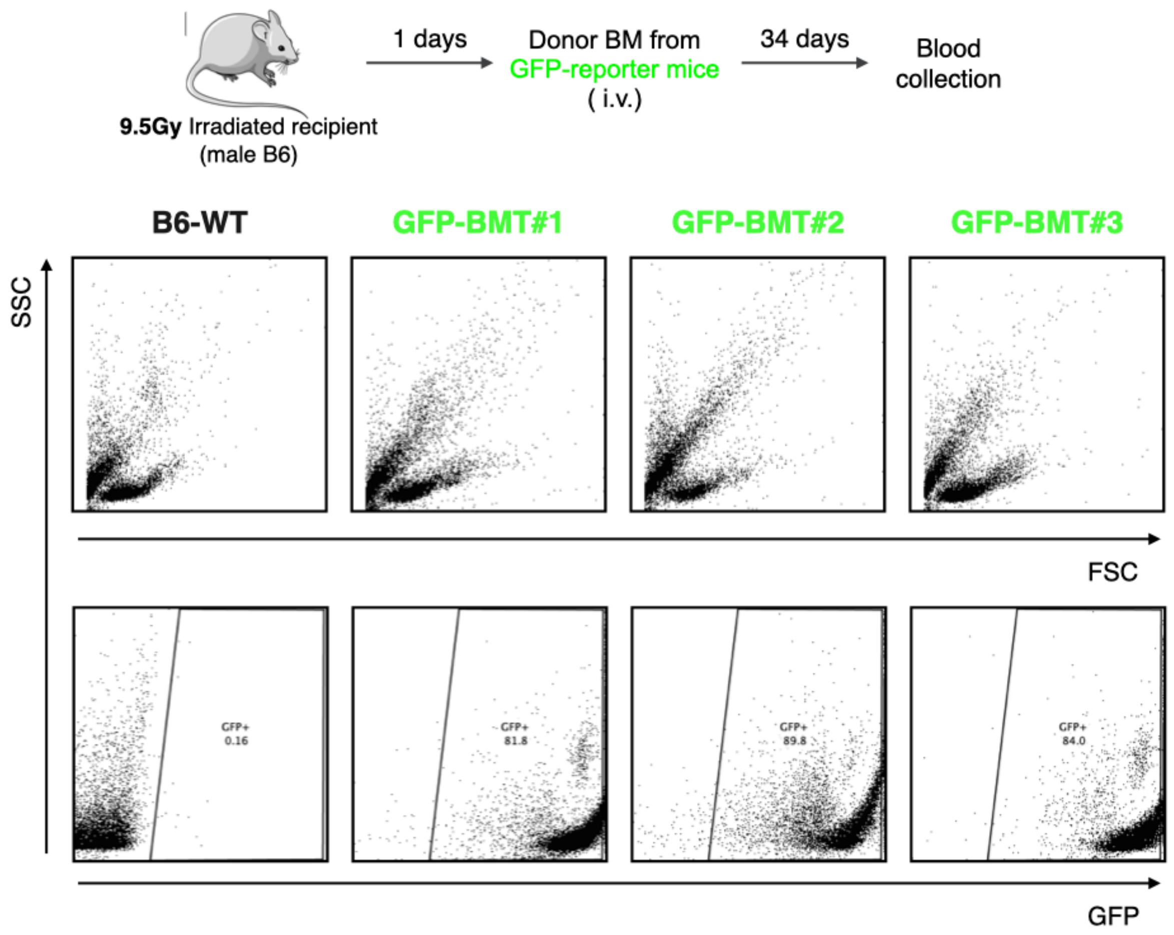

Supplementary Information Figure 7. GFP signal in peripheral blood of C57BL/6 mice after 34 days of BMT.

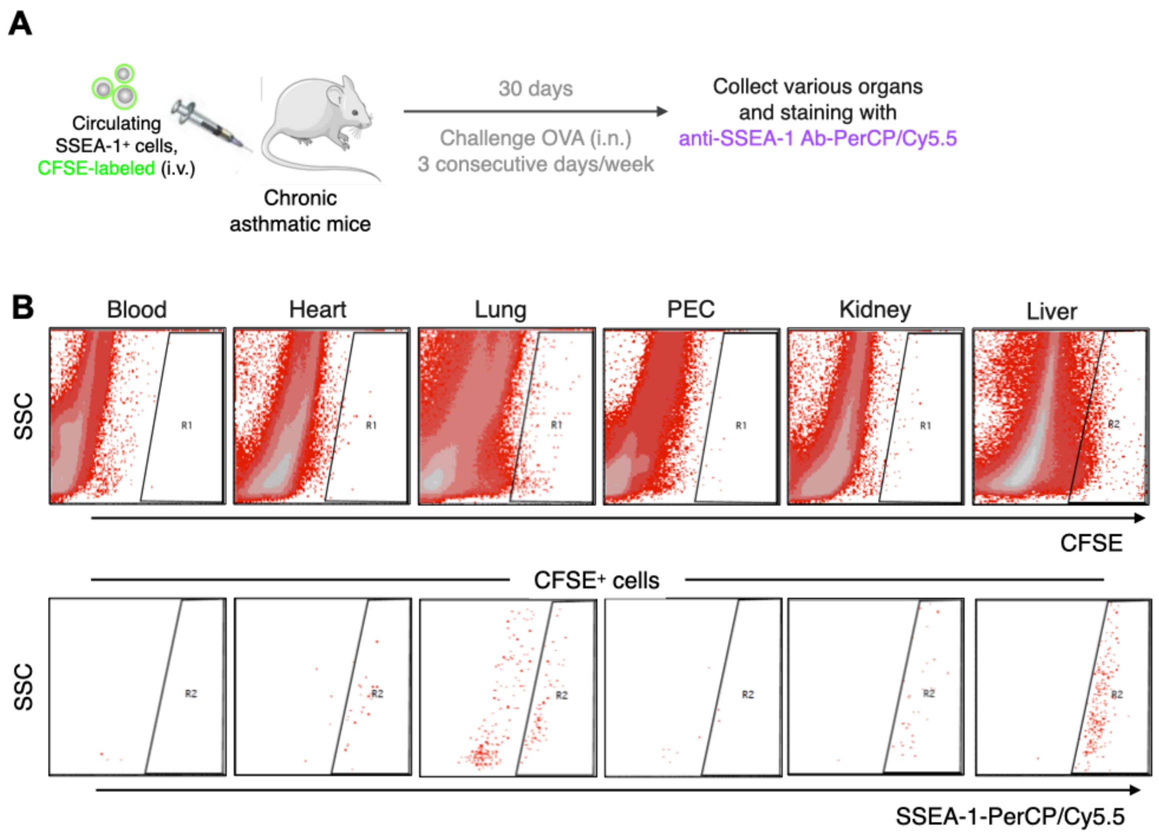

Supplementary Information Figure 8. Circulating SSEA-1$^+$ cells migrated into lung and loss its SSEA-1 marker. (A) Flowchart of the method used to track the SSEA-1 expression of transferred circulating SSEA-1$^+$ cells in chronic asthmatic mice. (B) SSEA-1 expression in adoptive transferred-CFSE cells. Enriched circulating SSEA-1$^+$ cells were labeled with CFSE and adoptively transferred into chronic asthmatic recipient mouse, followed by inhaled OVA challenge 3 times a week for 4 weeks. Thereafter, single cell suspensions from various organs were prepared and labeled with the same clone of PerCP/Cy5.5 conjugated-SSEA-1 antibody and analyzed by cytometry. CFSE$^+$ cells are gating in the box (R1). SSEA-1-PerCP/Cy5.5

analysis of CFSE cells gated in R1. The box R2 indicates SSEA-1$^+$ cells recovered from transferred CFSE$^+$ cells. Total 5×10^5 cells were acquired by FACS.

Supplementary Information Reference

Plantinga M, Guilliams M, Vanheerswynghels M, Deswarte K, Branco-Madeira F, Toussaint W, Vanhoutte L, Neyt K, Killeen N, Malissen B, Hammad H, Lambrecht BN (2013) Conventional and monocyte-derived CD11b(+) dendritic cells initiate and maintain T helper 2 cell-mediated immunity to house dust mite allergen. Immunity 38 (2):322-335. doi:10.1016/j.immuni.2012.10.016
